# Prokaryotic and eukaryotic microbiomes associated with blooms of the ichthyotoxic dinoflagellate *Cochlodinium (Margalefidinium) polykrikoides* in New York, USA, estuaries

**DOI:** 10.1371/journal.pone.0223067

**Published:** 2019-11-07

**Authors:** Theresa K. Hattenrath-Lehmann, Jennifer Jankowiak, Florian Koch, Christopher J. Gobler

**Affiliations:** Stony Brook University, School of Marine and Atmospheric Sciences, Southampton, NY, United States of America; Universitat Bremen, GERMANY

## Abstract

While harmful algal blooms caused by the ichthyotoxic dinoflagellate, *Cochlodinium (Margalefidinium) polykrikoides*, are allelopathic and may have unique associations with bacteria, a comprehensive assessment of the planktonic communities associated with these blooms has been lacking. Here, we used high-throughput amplicon sequencing to assess size fractionated (0.2 and 5 μm) bacterial (16S) and phytoplankton assemblages (18S) associated with blooms of *C*. *polykrikoides* during recurrent blooms in NY, USA. Over a three-year period, samples were collected inside (‘patch’) and outside (‘non-patch’) dense accumulations of *C*. *polykrikoides* to assess the microbiome associated with these blooms. Eukaryotic plankton communities of blooms had significantly lower diversity than non-bloom samples, and non-bloom samples hosted 30 eukaryotic operational taxonomic units (OTUs) not found within blooms, suggesting they may have been allelopathically excluded from blooms. Differential abundance analyses revealed that *C*. *polykrikoides* blooms were significantly enriched in dinoflagellates (*p*<0.001) and the experimental enrichment of *C*. *polykrikoides* led to a significant increase in the relative abundance of eight genera of dinoflagellates but a significant decline in other eukaryotic plankton. *Amoebophrya* co-dominated both within- and near- *C*. *polykrikoides* blooms and was more abundant in bloom patches. The core bacterial microbiome of the >0.2μm fraction of blooms was dominated by an uncultured bacterium from the SAR11 clade, while the >5μm size fraction was co-dominated by an uncultured bacterium from Rhodobacteraceae and *Coraliomargarita*. Two bacterial lineages within the >0.2μm fraction, as well as the Gammaproteobacterium, *Halioglobus*, from the >5μm fraction were unique to the microbiome of blooms, while there were 154 bacterial OTUs only found in non-bloom waters. Collectively, these findings reveal the unique composition and potential function of eukaryotic and prokaryotic communities associated with *C*. *polykrikoides* blooms.

## Introduction

Blooms of *Cochlodinium (*aka. *Margaelefidinium) polykrikoides* were first observed in Puerto Rico in the mid-20^th^ century [1], and have since been reported in regions across North America, Asia, Australia, and Europe [2]. Along the east coast of the United States, *C*. *polykrikoides* blooms were first reported in Narragansett Bay, Rhode Island, during the early1980s [3]. Over the past three decades, blooms have expanded to other regions along the US east coast, including the York River and Chesapeake Bay, Virginia [4,5], Skidaway Estuary, Georgia [6], Indian River Lagoon, Florida [7], multiple embayments across Long Island, New York [2,8–11], and Cape Cod, Massachusetts [12]. This ichthyotoxic dinoflagellate is well-known for causing fish kills across North America and Asia [1,2,13,14], causing significant financial losses (up to $100M) during bloom events [15–17]. Hence, there remains great interest in determining what causes these destructive blooms to recur and expand on a global scale.

Like many other harmful dinoflagellates, *C*. *polykrikoides* has a number of ecological strategies that aid in bloom development and maintenance, allowing it to outcompete other phytoplankton. Among these strategies, diverse nutrient acquisition capabilities [8,18], including mixotrophy [19,20], grazer inhibition [2,21,22] and the production of allelochemicals that lyse or inhibit the growth of competing phytoplankton [23], have been elucidated. Recently, the ability of *C*. *polykrikoides* to produce resting cysts in culture was confirmed [24] and cysts have been identified in sediments around Long Island, NY [25] as well as Korea [26,27]. Furthermore, decadal ocean warming trends on the US east coast and in eastern Asia have increased the growth rates of *C*. *polykrikoides* and expanded the bloom season of strains from these regions by more than a month [28]. Beyond bloom development and maintenance, microbial associations may influence the recurrence and expansion of *C*. *polykrikoides* blooms.

There have been a limited number of studies assessing bacterial associations and interactions with *C*. *polykrikoides*. *C*. *polykrikoides* is capable of grazing on bacteria [20] and bacteria capable of lysing this dinoflagellate have been described [29–31]. Using terminal restriction fragment length polymorphism analysis of 16S rRNA genes Koch et al. [9] described significant differences in bacterial community composition between *C*. *polykrikoides* bloom (patch) and non-bloom (non-patch) samples in NY, although the method used, prohibited the identification of the microbes responsible for these differences. Park et al. [32] assessed bacterial community dynamics during *C*. *polykrikoides* blooms in Korea using clone library analysis and found Rhodobacterales increased and gamma-proteobacteria decreased in abundance during *C*. *polykrikoides* blooms. Clone libraries, however, can be biased and often do not detect rare microbes [33–35]. Recently, there have been a growing number of studies utilizing amplicon-based high-throughput sequencing to assess the microbial consortia associated with HABs [36–41], an approach that provides a significant advance in resolution of taxa relative to past effort [33–35,42]. Two recent studies used high-throughput sequencing to identify bacteria associated with *C*. *polykrikoides* cultures isolated from Korea [40,41], however, there are currently no studies using this technology to determine the microbiome associated with blooms of *C*. *polykrikoides*.

Here, we used high-throughput amplicon sequencing to assess bacterial (16S) and phytoplankton assemblages (18S) associated with blooms of the ichthyotoxic dinoflagellate, *C*. *polykrikoides*, during recurrent blooms in two estuaries on eastern Long Island, NY, USA. Over a three-year period, samples were collected inside (‘patch’) and outside (‘non-patch’) dense accumulations of *C*. *polykrikoides* to determine the core bacterial and eukaryotic microbiomes associated with these blooms. Size fractionation was used to describe and compare free-living (>0.2μm) and potential epiphytic or intracellular (>5 μm) bacterial assemblages associated with *C*. *polykrikoides* blooms. In addition, cultured *C*. *polykrikoides* cells were added to the natural plankton community to assess allelopathic effects on planktonic assemblages. This study revealed the presence of a parasitic dinoflagellate, *Amoebophrya* spp., as well as a number of novel bacteria associated with *C*. *polykrikoides* blooms.

## Materials and methods

### Study site sampling

Field samples were collected in 2011, 2012, 2013 and 2014 (field experiments) during *C*. *polykrikoides* blooms in Shinnecock Bay (40.860881N, 72.470632W) and Great Peconic Bay (40.938729N, 72.515285W), New York, which have occurred annually since 2004 [10]. Shinnecock Bay is a shallow, well-mixed system that exchanges with the Atlantic Ocean through the Shinnecock Inlet. Great Peconic Bay is a shallow, well-mixed system located between Long Island’s north and south forks and exchanges with the Atlantic Ocean. All samples were collected between 10:00am and 12:00pm. Sample collection did not involve any protected or endangered species and did not require any specific permits as samples were collected in regions open to the public. At each sampling point, samples were first collected within the visually dense, darkly-colored bloom patches (>1,000 *C*. *polykrikoides* cells mL^-1^) and then in regions adjacent to patches where the water was not discolored (<100 *C*. *polykrikoides* cells mL^-1^), designated ‘patch’ and ‘non-patch’ samples, respectively. For molecular analysis, patch and non-patch water was filtered onto 0.2*μ*m and 5*μ*m polycarbonate filters and immediately frozen at -80°C.

### DNA extraction, illumina sequencing and analysis

To extract nucleic acids, 1 mL of cetyltrimethyl ammonium bromide (CTAB) buffer with fresh beta-mercaptonethanol was added to the 0.2*μ*m and 5*μ*m polycarbonate filters, vortexed, heated to 50°C for 20 minutes, and frozen at -80°C until processing. Genomic DNA extraction was performed using the CTAB method [43]. Following extraction, double-stranded DNA was quantified on a Qubit® fluorometer using a dsDNA BR Assay kit. Alongside samples, 0.2μm and 5μm filter controls were extracted and DNA quantified (dsDNA = 0 ng/μl) as above to ensure that sample sequences were not a result from contamination during sample processing. Samples were normalized to an equal quantity of DNA for sequencing and were sent to Molecular Research Labs (Shallowater, Texas, USA) for amplicon sequencing. The 16S rRNA gene V4 variable region (~300bp) was amplified using bacterial primers A519F: 5´CAG CMG CCG CGG TAA and 802R: 5´TAC NVG GGT ATC TAA TCC [44]. The V7/V8 region of the 18S rRNA gene (~450bp) was amplified using primers 1183F: 5´AAT TTG ACT CAA CAC GGG and 1631aR: 5´TAC AAA GGG CAG GGA CG [45]. For each sample, an identifying barcode was placed on the forward primer and a 30 cycle PCR using the HotStarTaq Plus Master Mix Kit (Qiagen, USA) was performed. The following PCR conditions were used: 94°C for 3 minutes, followed by 28 cycles of 94°C for 30 seconds, 53°C for 40 seconds and 72°C for 1 minute, and a final elongation step at 72°C for 5 minutes. Successful amplification was determined by visualizing PCR products using a 2% agarose gel. The amplification of filter controls did not result in any products and therefore were not sequenced. Samples (31 for 18S and 24 for 16S, S1 and S2 Tables, respectively) were pooled together for each respective primer region in equal proportions based on their molecular weight and DNA concentrations. Pooled samples were then purified using calibrated Ampure XP beads and subsequently used to prepare a DNA library by following Illumina TruSeq DNA library. Paired-end (2x300) sequencing was performed on an Illumina MiSeq following the manufacturer’s guidelines. Sequence data was processed using the Quantitative Insights Into Microbial Ecology v1.9.1 pipeline (QIIME, http://qiime.org [46]). Raw sequences were depleted of barcodes, paired-end reads joined, depleted of primers, demultiplexed, and quality filtered using the default parameters in QIIME. The resulting quality filtered sequences were then clustered into operational taxonomic units (OTUs) at 97% similarity with UCLUST [47] using the open reference clustering protocol and SILVA release v119 (http://www.arb-silva.de/) as the reference set. The representative sequence set was aligned using PyNAST [48] and taxonomically classified using UCLUST [47]. For 18S specifically, all non-algal OTUs were not considered in order to focus specifically on algal assemblages. Further, the classes Dinophyceae and Syndiniophyceae were considered as a single group representing total dinoflagellates and from here on are referred to as Dinophyceae. Since species specificity with the QIIME pipeline was typically not possible, representative sequences for the most abundant OTUs were extracted and species specificity (percent identity) was determined using BLAST. Similarly, for 16S, since our focus was on prokaryotes, all chloroplast and mitochondria (S2 Table) related sequences were removed from OTU tables and not further considered in analyses. In addition, OTUs identified as *Prochlorococcus* were reassigned to *Synechococcus* II as another distinct group assigned as *Synechococcus* was already identified within our dataset [49]. NCBI BLAST results supported this reassignment as ‘*Prochlorococcus*’ OTU consensus sequences were 100% identical to *Synechococcus* sp. MV0605E (accession ID KU867943.1). Furthermore, phylogenetic analysis of sequences from the informatically identified *Prochlorococcus* and *Synechococcus* suggests that they are an intermixed group (data not shown). Finally, the ecology of these two organisms further supports this reassignment as *Prochlorococcus* is absent from eutrophic coastal regions including estuaries whereas *Synechococcus* inhabits a broader niche including eutrophic estuaries [50].

For the V7/V8 region of the 18S rDNA, the 30 samples generated 3,802,417 paired end reads with an amplicon size of ~450bp. After quality filtering and joining reads a total of 3,345,085 reads clustered at 97% identity into 41,828 OTUs. Overall, algae represented 24 to 97% of total reads (prior to removal of non-algal OTUs) with an average of 78% for all 30 samples (S1 Table). For the 519F/802R region of 16S rDNA, the 24 samples generated 3,951,820 paired end reads with an amplicon size of ~300bp. After quality filtering and joining reads a total of 3,617,344 reads clustered at 97% identity into 16,905 OTUs (S2 Table). The 870,170 and 4,570 reads assigned as chloroplasts and mitochondria, respectively, were removed from the dataset and not considered in analyses (S2 Table).

### Culture and culturing conditions

A locally isolated culture of *C*. *polykrikoides* (CP1, isolated from the Peconic Estuary, NY; [10]) was used for this study. CP1 was cultivated in sterile GSe medium [51] with a salinity of 32 PSU, made with autoclaved and 0.2 μm-filtered aged coastal Atlantic Ocean water (40.79698N, 72.46068W), at 21°C in an incubator with a 12:12 h light:dark cycle, illuminated by a bank of fluorescent lights that provided a light intensity of ~100 μmol quanta m^-2^ s^-1^ to cultures. Antibiotics (stock solution, Thermo Scientific HyClone Penicillin (10,000U mL^-1^) Streptomycin (10,000μg mL^-1^) in 0.85% NaCl) were added to the CP1 culture at a final concentration of 1% by volume to discourage microbial contamination.

### Allelopathy experiment

An experiment was performed to assess the natural plankton community’s response to the addition of environmentally relevant densities of *C*. *polykrikoides*. While the allelopathic effects of *C*. *polykrikoides* on natural phytoplankton communities and cultures have been assessed microscopically [23], the effects on smaller phytoplankton and bacterial communities have never been examined. During October 2014, triplicate 330mL bottles were half-filled with unamended water from Old Fort Pond, NY. A control was established whereby the other half of the bottle was filled with GSe media and, for the treatment, the other half of the bottle was filled with *C*. *polykrikoides* (CP1) culture resulting in final cell densities of 2,200 cells mL^-1^. To ensure that the effects seen by the addition of *C*. *polykrikoides* were due to allelochemicals and not nutrients, saturating concentrations of N (88μM), P (3.6μM), and Si (88μM) were added to all bottles. All experimental bottles were incubated at ambient light and temperature for 48 h in Shinnecock Bay at the Stony Brook Southampton Marine Science Center [52]. At the end of the incubation, contents of each experimental bottle were filtered onto 0.2μm polycarbonate filters that were then preserved as above and sequenced, in triplicate, as individual biological replicates.

### Microbial diversity and statistical analysis

All microbial diversity analyses were conducted using QIIME v1.9.1. Prior to executing diversity analysis scripts on field samples, mitochondrial and chloroplast related OTUs as well as all non-algal OTUs were filtered from 16S and 18S OTU tables, respectively. Field sample datasets were then processed using core_diversity_analyses.py scripts to rarefy samples and assess alpha- (Chao1, Shannon Diversity Index and Simpson Index) and beta- (weighted unifrac visualized via Principal Coordinates Analysis (PCoA)) diversity measurements. An Analysis of Similarity (ANOSIM) was conducted using weighted unifrac distances to assess the differences in community composition among variables.

In addition, the core microbiome of field samples for both 16S and 18S datasets were computed for each variable (patch >0.2μm, patch >5μm, non-patch >0.2μm, and non-patch >5μm) using compute_core_microbiome.py scripts in QIIME and visualized using Venny 2.0 [53]. Core microbiomes were defined as genera found in 100% of the samples within each variable (as above). Differential abundance analyses were also conducted, using the Phyloseq and DESeq packages [54–56] in R v3.2.3 (R Core Team 2013) to compare taxa abundances across experimental treatments as well as field sample variables (patch >0.2μm vs non-patch >0.2μm, and patch >5μm vs non-patch >5μm) for both 16S and 18S data. Briefly, raw 16S and 18S read counts were loaded into a phyloseq object and modeled with Deseq using a negative binomial distribution. Prior to modeling, counts were internally normalized for size factors using the median ratio method [54] and dispersions were estimated using a parametric fitting. Wald significance testing to test for significant log2 fold changes in abundance (α = 0.05) and p-values were adjusted using the Benjamini-Hochberg procedure to correct for multiple testing. Principal component analyses (PCA) were conducted on Hellinger transformed ($y^{\prime }ij=\surd \frac{yij}{yi+}$) abundances of bacterial sequences [57] using the prcomp function (stats package) in R v3.3.3 to determine the groups of variables (region: patch, non- patch; size fraction: >0.2 and >5μm; year: 2011, 2012, 2013) that behaved similarly and the correlation of taxa (phylum or genus) to each group (biplot arrows).

To investigate potential functional differences among the microbial communities between regions, size fractions and years, predicted metagenomes were generated using PICRUST2 (Phylogenetic Investigation of Communities by Reconstruction of Unobserved States) QIIME2 plugin with default settings [58–62]. Prior to analysis the 16S OTU abundances were normalized (relative abundances) and the resulting predicted KEGG KO gene family and Metacyc pathway abundances were visualized with PCoA (QIIME2 Version 2019.1). PERMANOVA (QIIME2 diversity plugin) was used to test for significant differences among the predicted metagenomes between groups (region, size fraction, year) and was used to identify differentially abundant gene families and pathways among groups. Differential abundance of KEGG gene families and Metacyc pathways were assessed using STAMPS v 2.1.3 software [63] with a multiple sample ANOVA and Tukey-Kramer post-hoc analysis with Benjamini-Hochberg FDR multiple comparison correction. Genes and pathways were filtered for p<0.05 and an eta-squared effect size of >0.8.

## Results

### Phytoplankton assemblages within- and outside- of *C*. *polykrikoides* patches

**Fig 1:**
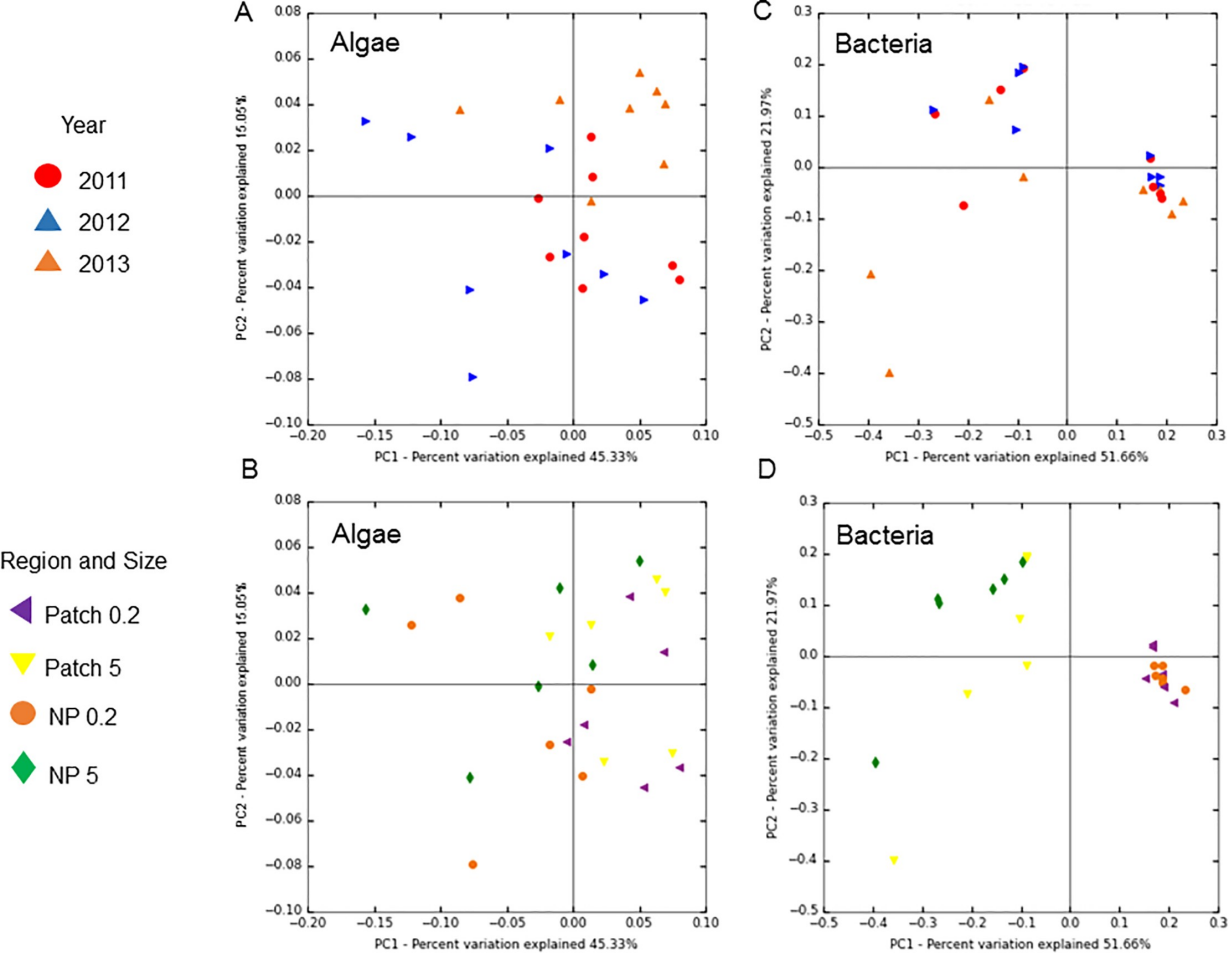
Principal Coordinate Analysis (PCoA) using weighted UniFrac distances of A & B) algal sequences and C & D) bacterial sequences for all field samples by year (2011, 2012 and 2013), and region (patch vs non-patch) and size fraction (>0.2 and >5 micron).

An Analysis of Similarity (ANOSIM) revealed significant differences in the community composition among patch and non-patch (*p*<0.001) samples, and among years (*p*<0.001) but not among size fractions. All alpha diversity metrics revealed no significant differences among size class (>0.2 or >5μm) or year (2011, 2012 and 2013) but indicated non-patch samples were significantly (*p*<0.01) more diverse than within patch samples (S3 Table). A principal coordinates analysis (PCoA) of weighted unifrac distances showed clustering among years (Fig 1A) and sample type (patch, non-patch; Fig 1B) with the diversity between these variables being significantly (*p*<0.05) different.

**Fig 2:**
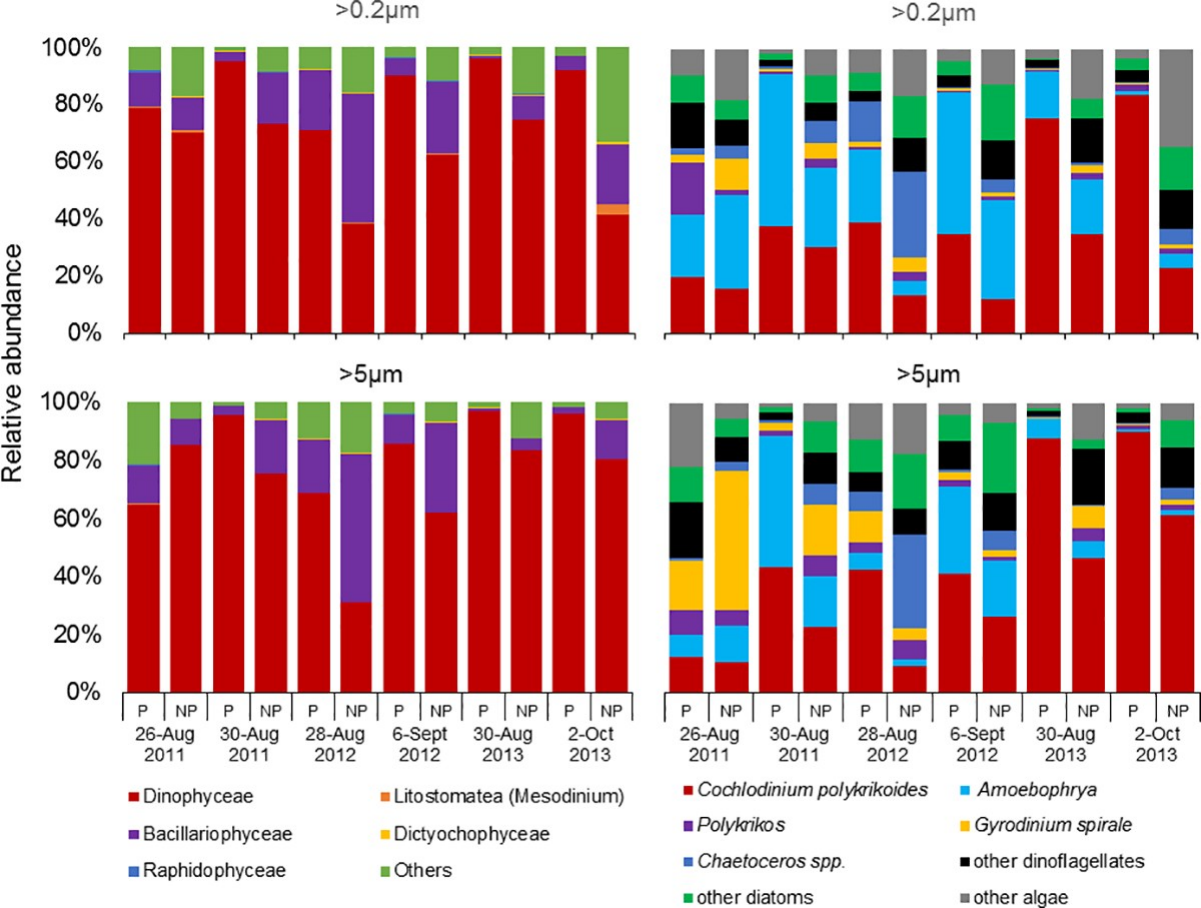
Class- (left panel) and genus/species- (right panel) level relative abundances of algal sequences from size fractionated (>0.2 and >5 micron) samples collected within and immediately adjacent (<10m) to the *Cochlodinium* bloom patches, designated ‘patch’ (P) and ‘non-patch’ (NP) samples, respectively, for years 2011, 2012 and 2013.

While differential abundance analyses revealed that Dinophyceae (dinoflagellates) was significantly (*p*<0.01; S1 Fig) enriched within *C*. *polykrikoides* patches, it was also considered the dominant phyla in all samples, contributing 86 ± 12% (mean ± SD) of algal sequences within patches, and 65 ± 18% outside of patches, respectively (Fig 2). Bacillariophyceae (diatoms; *p* = 0.15) was enriched in non-patch (21 ± 14%) communities (>5μm size fraction only), compared with patch communities (8 ± 7%), but not significantly (S1 Fig). Both the dominant genera in all samples and considered enriched in patch samples, *C*. *polykrikoides* (*p*<0.001; S2 Fig) and *Amoebophrya* (p = 0.09) contributed 51 ± 27% and 22 ± 19% within patches, and 26 ± 16% and 15 ± 12% outside of patches (Fig 2). However, for the >0.2μm size fraction of patches, while the range of relative abundances were higher for *C*. *polykrikoides*, *Amoebophrya* often had a higher relative abundance on a per sample basis, especially during 2011 and 2012 (Fig 2). *Gyrodinium* and *Chaetoceros* were also found at modest abundances (<48%) but overall were more abundant outside of *C*. *polykrikoides* patches (Fig 2).

**Fig 3:**
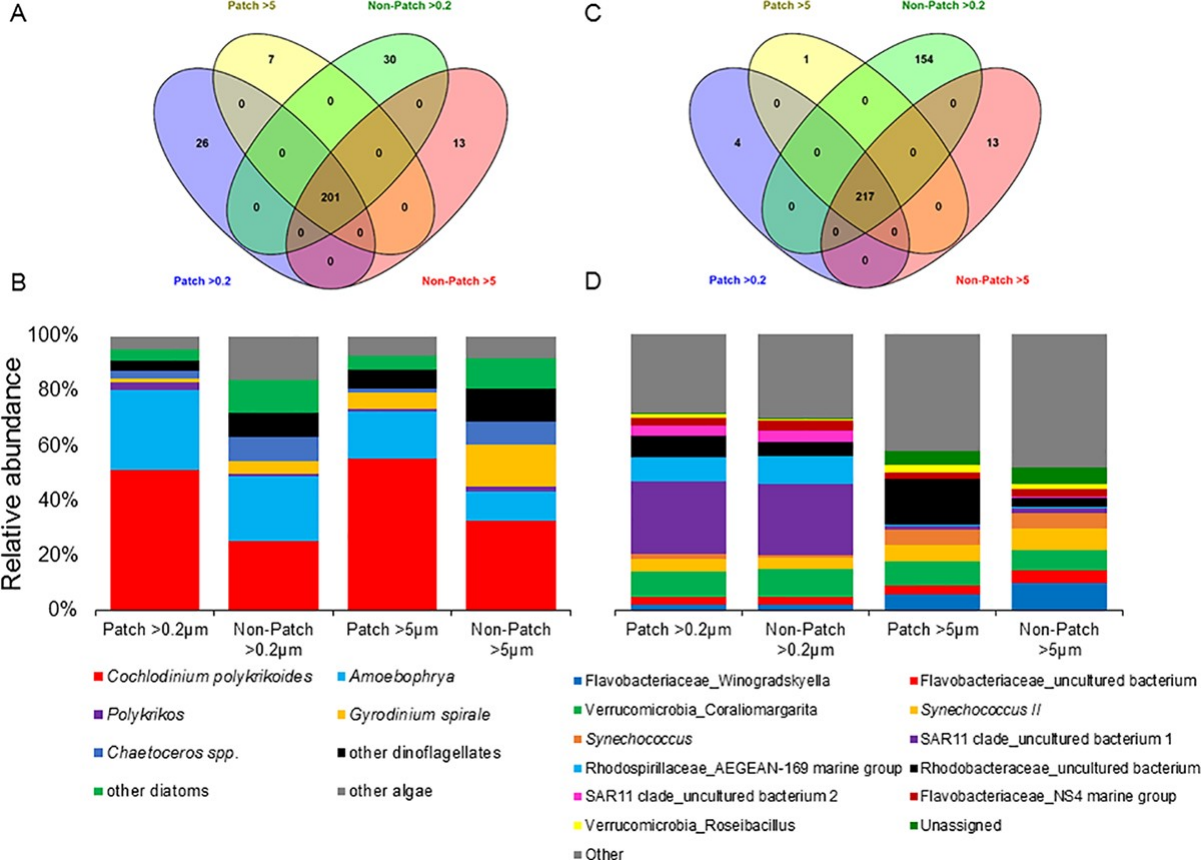
Venn diagrams demonstrating the shared and unique A) 18S OTUs-, C) 16S OTUs- and average abundances of corresponding B) 18S consensus lineages- and D) 16S consensus lineages- of the core microbiomes found among Patch >0.2 and >5, and Non-patch >0.2 and >5 micron size fractioned samples. Venn diagrams colors represent the following: patch >0.2 (blue), patch >5 (yellow), non-patch >0.2 (green) and non-patch >5 (red). Numbers within the diagrams represent number of OTUs.

Comparing the core 18S microbiomes among each variable (patch >0.2μm, patch >5μm, non-patch >0.2μm, and non-patch >5μm), there were 201 shared OTUs and 7–30 unique OTUs (Fig 3A). The core 18S microbiome for all variables was dominated by *C*. *polykrikoides* (>25%) and *Amoebophrya* (>11%; Fig 3B). Among these microbiomes, there were 26 (from four lineages) and 30 (eight lineages) unique OTUs from patch >0.2μm and non-patch >0.2μm samples, and 7 (one lineage) and 13 (one lineage) unique OTUs for patch >5μm and non-patch >5μm samples, respectively (Fig 3A). Unique OTUs associated with patch samples included lineages of *Geminigera* sp., *Cylindrotheca closterium*, *Leptocylindrus* spp. and an uncultured eukaryote from MAST-3J, and *Pirsonia* sp. for the >0.2μm and >5μm size fractions, respectively. Unique OTUs associated with non-patch samples included lineages of *Gonyaulax* sp., *Rhaphoneis* sp. (Bacillariophyceae), two cryptophytes (*Katablepharis* sp., *Leucocryptos* sp.), two Prymnesiales (uncultured eukaryote and OLI16029), and two uncultured eukaryotes (Mamiellophyceae and Chrysophyceae), and an uncultured eukaryote from the Syndiniales for the >0.2μm and >5μm size fractions, respectively.

### Bacterial assemblages within- and outside- of *C*. *polykrikoides* patches and between size fractions

**Fig 4:**
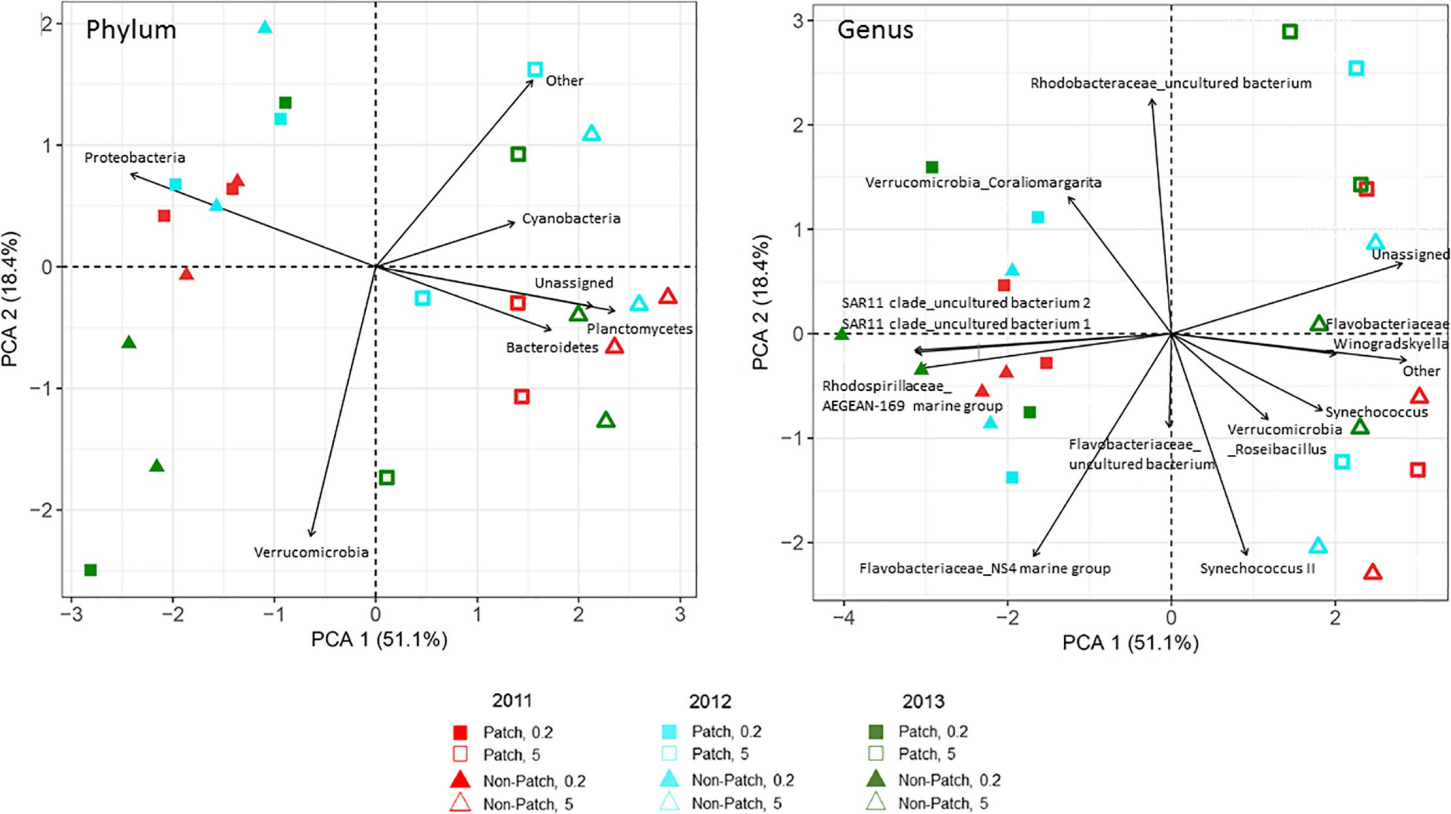
Principal component analyses using Phylum- and genus/species- level Hellinger transformed relative abundances of bacterial sequences from size fractionated (>0.2 and >5 micron) samples collected within and immediately adjacent (<10m) to the *Cochlodinium* bloom patches, designated ‘patch’ (P) and ‘non-patch’ (NP) samples, respectively, for years 2011, 2012 and 2013. Percent variation explained by each principal component is indicated in parentheses.

The community composition of bacterial assemblages among the patch and non-patch samples were similar (ANOSIM; *p*>0.05) while the community composition between size fractions (>0.2 vs >5μm) was significantly different (ANOSIM; *p*<0.001). Similarly, alpha diversity metrics revealed significant differences among size class (>0.2 or >5μm) with the >5μm size fraction being significantly (*p*<0.01) more diverse than the >0.2μm size fraction (S4 Table). A principal coordinates analysis (PCoA) of weighted unifrac distances displayed a high degree of clustering among size fractions (>0.2 and >5μm; Fig 1C and 1D), with the diversity between these variables being highly significantly (*p*<0.001) different. Consistent with this, principle component analyses (PCA) of relative abundances at both phylum- and genus/species- level revealed that samples separated based on size fraction (>0.2 and >5μm; Fig 4). At the phylum level, the >0.2μm fraction was highly associated with Proteobacteria, while the >5μm was associated with Planctomycetes and Bacteroidetes (Fig 4). At the genus/species level, the >0.2μm fraction was highly associated with two uncultured bacteria from the SAR11 clade and the AEGEAN-169 marine group, while the >5μm was more associated with *Synechococcus* and the Flavobacteriaceae, *Winogradskyella* (Fig 4).

**Fig 5:**
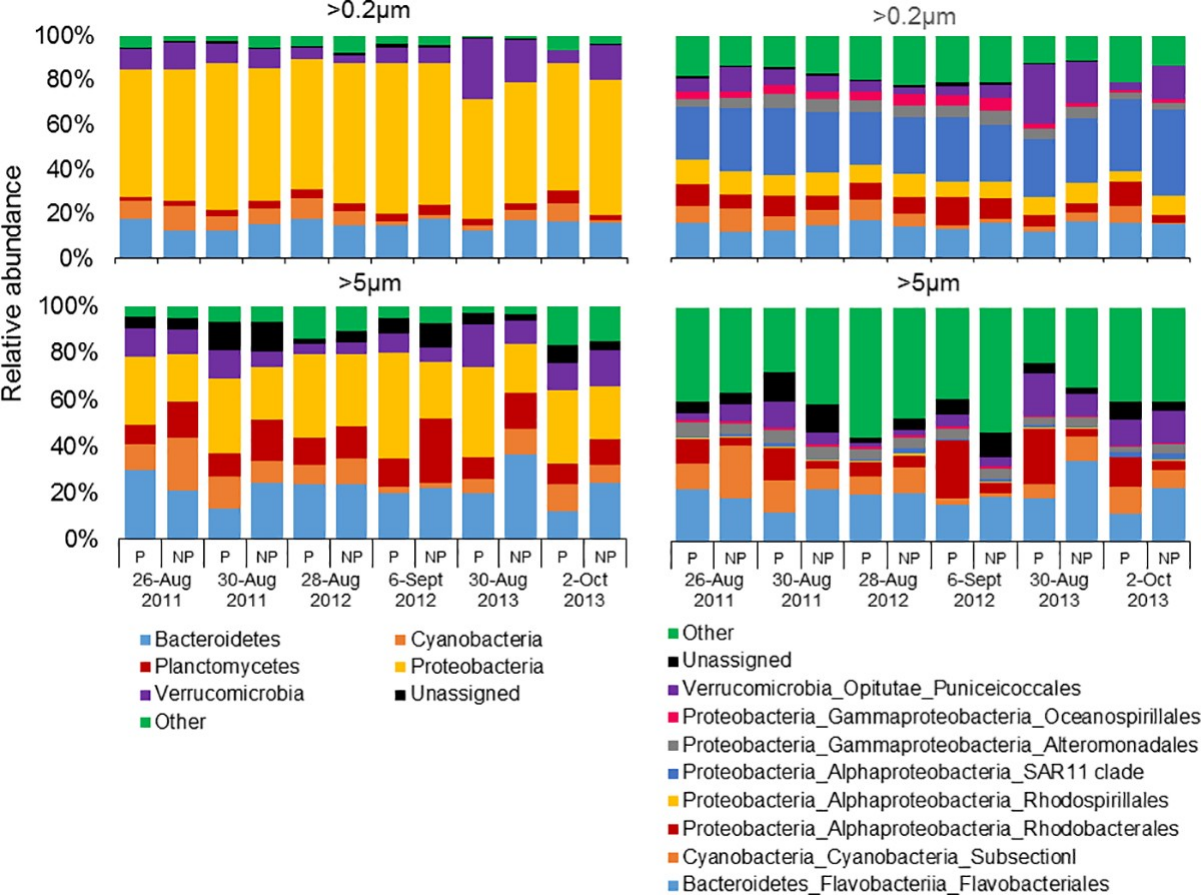
Phylum- (left panel) and Order- (right panel) level relative abundances of bacterial sequences from size fractionated (>0.2 and >5 micron) samples collected within and immediately adjacent (<10m) to the *Cochlodinium* bloom patches, designated ‘patch’ (P) and ‘non-patch’ (NP) samples, respectively, for years 2011, 2012 and 2013.

**Fig 6:**
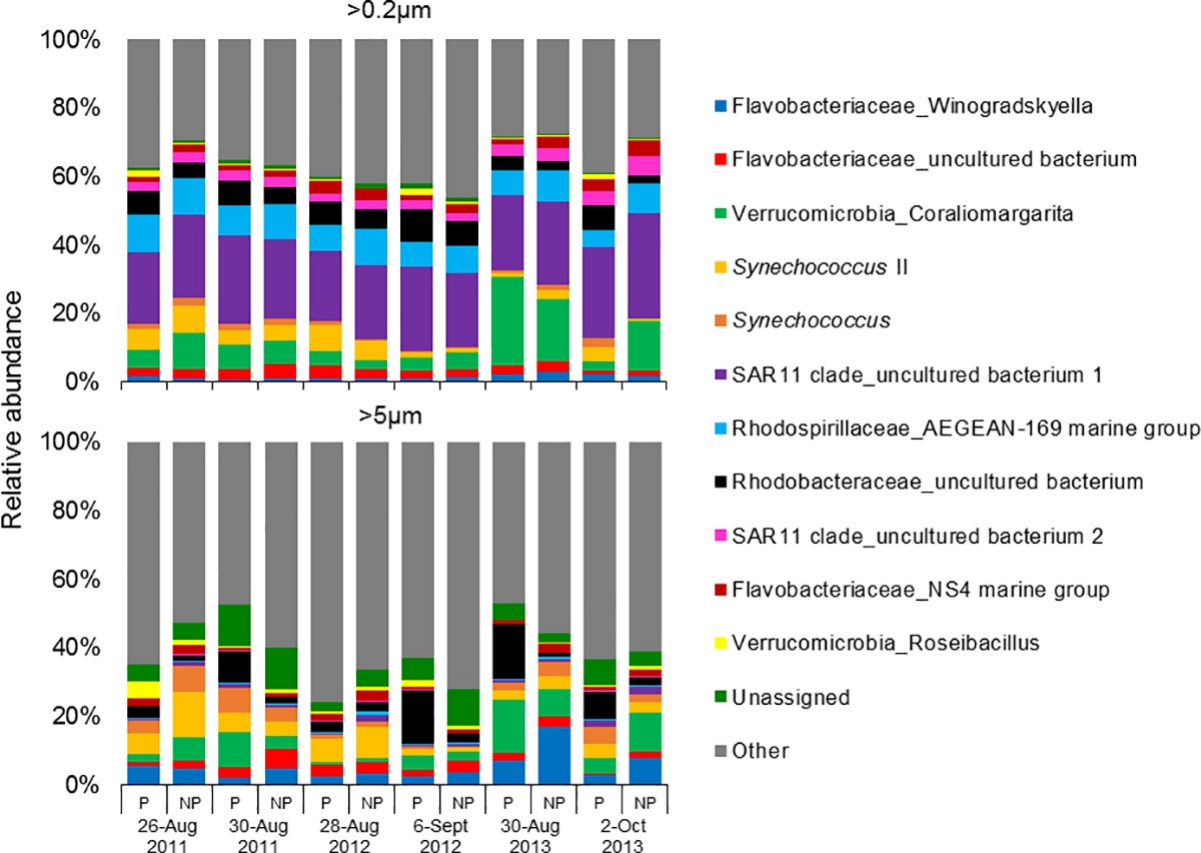
Genus- level relative abundances of bacterial sequences from size fractionated (>0.2 and >5 micron) samples collected within and immediately adjacent (<10m) to the *Cochlodinium* bloom patches, designated ‘patch’ (P) and ‘non-patch’ (NP) samples, respectively, for years 2011, 2012 and 2013.

While patch and non-patch samples were similar there were vast differences between size fractions. Proteobacteria was the dominant phyla followed by Bacteroidetes contributing 60 ± 4% (mean ± SD) and 16 ± 2% of bacterial sequences for the >0.2μm fraction, and 30 ± 8% and 23 ± 7% for the >5μm size fraction, respectively (Fig 5). The SAR11 clade (28 ± 4%) and Flavobacteriales (14 ± 2%) dominated bacterial sequences in >0.2μm fraction, while for the >5μm fraction, the orders Flavobacteriales (20 ± 6%), Cyanobacteria Subsection 1 (which includes *Synechococcus;* 10 ± 5%), and Rhodobacterales (10 ± 7%) dominated bacterial sequences (Fig 5). An uncultured bacterium (identified as ‘1’ in Fig 6) from the SAR11 clade (24 ± 3%) was the most dominant genus found in the >0.2μm fraction followed by a bacterium from the AEGEAN-169 marine group (9 ± 2%) and *Coraliomargarita* (9 ± 7%; Fig 6). At the genus level, the >5μm fraction was dominated by sequences of *Coraliomargarita* (6 ± 4%), an uncultured bacterium from Rhodobacteraceae (6 ± 5%) and *Winogradskyella* (5 ± 4%; Fig 6).

#### Comparing size fractions within patch and non-patch communities

These differences among size fractions are further highlighted when comparing size fractions within patch and non-patch communities. Within *Cochlodinium* patches, differential abundance analyses demonstrated that the SAR11 clade, Rhodospirillales, and Oceanospirillales were all significantly (*p*<0.001) enriched in the >0.2μm compared to the >5μm fraction (S3A Fig). Among the most abundant genera, two uncultured bacteria (identified as ‘1’ and ‘2’ in Fig 6) of the SAR11 clade and the AEGEAN-169 marine group were significantly (p<0.001) enriched in the >0.2μm fraction of patches, while *Winogradskyella* and the cyanobacteria *Synechococcus* were significantly (p<0.01) enriched in the >5μm fraction (S4A Fig). Comparing size fractions within patch communities for all genera, there were 274 lineages that were significantly differentially abundant, with 88 enriched in the >0.2 size fraction and 186 enriched in the >5μm size fraction (p<0.05 for all; S6–S13 Figs).

For non-patch communities, differential abundance analyses demonstrated that the SAR11 clade, Rhodospirillales and Oceanospirillales were significantly (p<0.001) enriched in the >0.2μm fraction, while Flavobacteriales and Cyanobacteria Subsection 1 were significantly (p<0.01) enriched in the >5μm fraction (S3B Fig). Among the most abundant genera, two uncultured bacteria (1, 2) of the SAR11 clade and AEGEAN-169 marine group were significantly (p<0.001) enriched in the >0.2μm fraction of non-patch samples, while *Winogradskyella* and the cyanobacteria *Synechococcus* were significantly (p<0.01) enriched in the >5μm fraction (S4B Fig). Comparing size fractions within non-patch communities for all genera, there were 259 lineages that were significantly differentially abundant, 103 and 156 of those lineages were enriched in the in the >0.2 and >5μm size fractions, respectively (S14–S21 Figs).

#### Patch vs non-patch within the >0.2μm fraction

Differential abundance analyses of major microbial orders and genera (i.e. taxonomic orders and genera with the highest relative abundances in Figs 5 and 6) revealed there were no significant differences between the patch and non-patch samples within the >0.2μm fraction (S3C and S4C Figs). Comparing patch and non-patch samples within the >0.2μm fraction for all genera, however, revealed that 2 lineages (Thermoplasmatales Marine Group II uncultured and Thermoplasmatales Marine Group II other) were considered significantly enriched in non-patch samples, while uncultured Acidimicrobiaceae was significantly enriched in patches (p<0.01 for all; S5A Fig).

#### Patch vs non-patch within the >5μm fraction

Among the >5μm fraction, differential abundance analyses demonstrated that the order Rhodobacterales was significantly (*p*<0.001) enriched in patch samples while the orders Rhodospirillales and Flavobacteriales were significantly (p<0.01) enriched in non-patch samples (S3D Fig). As for the major (i.e. most abundant) genera in the >5μm fraction, an uncultured bacterium from Rhodobacteraceae was significantly enriched in patch samples (*p*<0.001; S4D Fig). Comparing patch and non-patch samples within the >5μm fraction for all genera, however, revealed two lineages (uncultured *Rictus* and Stramenopiles MAST-12D other) that were significantly enriched in non-patch samples and 19 lineages (including a number of Alphaproteobacteria and Bacteroidetes) were significantly enriched in the patch samples (p<0.05 for all; S5B Fig).

Comparing the microbiomes among each sample type, there were 217 shared OTUs, and 4, 1, 154 and 13 unique OTUs for patch >0.2μm, patch >5μm, non-patch >0.2μm, and non-patch >5μm, respectively (Fig 3C). The core bacterial microbiome was dominated by an uncultured bacterium (designated as ‘1’ in Fig 6) from the SAR11 clade for patch and non-patch samples in the >0.2μm fraction, and overall looked very similar (Fig 3D). For the >5μm size fraction, the patch microbiome was co-dominated by an uncultured bacterium from Rhodobacteraceae and *Coraliomargarita* (Verrucomicrobia), while the non-patch samples were co-dominated by *Winogradskyella* (Flavobacteriaceae), the cyanobacteria *Synechococcus* (type *II*) and *Coraliomargarita* (Fig 3D). Among these microbiomes, there were four and 154 unique OTUs for the patch and non-patch samples in the >0.2μm fraction, respectively, originating from two (an unassigned bacterium, and an uncultured bacterium from Rickettsiales) and 66 unique lineages, respectively. There were one and 13 unique OTUs for the patch and non-patch samples in the >5μm fraction, respectively, originating from one (the Gammaproteobacteria, *Halioglobus*) and seven unique lineages, all of which, however, had relative abundances of <1%.

### The natural plankton community’s response to the addition of *C*. *polykrikoides*

**Fig 7:**
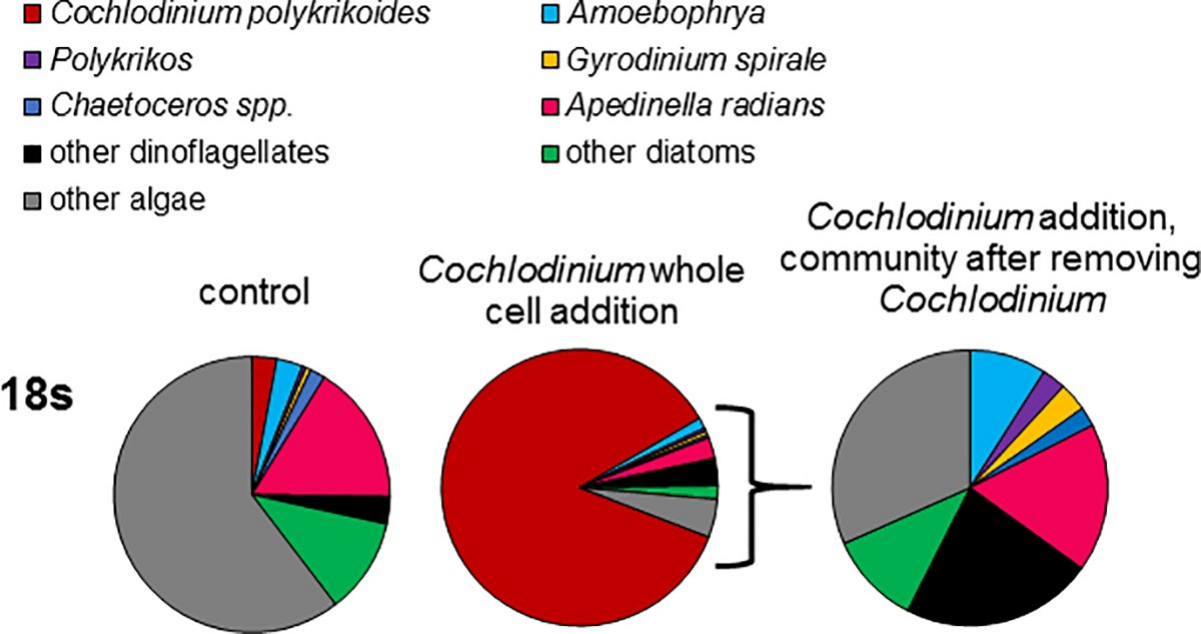
Relative abundances of algal (18S) sequences for the experimental control, and the *Cochlodinium polykrikoides* culture whole cell addition treatment during an experiment conducted using the natural phytoplankton community of Old Fort Pond, NY.

The addition of a culture of *C*. *polykrikoides* resulted in marked changes in the relative abundances of the eukaryotic plankton (Fig 7). Differential abundance analyses between the control and treatment revealed that eight dinoflagellates were significantly enriched with the addition of *C*. *polykrikoides* including *Amoebophrya* (*p*<0.001), *Gyrodinium spirale* (*p*<0.001), *Polykrikos* (*p*<0.001), *Karlodinium* (*p*<0.001), *Prorocentrum* (*p*<0.001), *Symbiodinium* (*p*<0.001), *Gonyaulax* (*p*<0.05), and *Pelagodinium* (*p*<0.001; S22 Fig). While the relative abundances of *Chaetoceros* and *Apedinella* also increased in the treatment (Fig 7), the addition of *C*. *polykrikoides* caused ‘other algae’ to decrease by nearly 50% compared to the control (Fig 7) and caused the significant (*p*<0.05) decline of a number of cryptomonads, chrysophytes and other non-dinoflagellate genera (S22 Fig). Due to the mode in which allelopathic agents are secreted by *C*. *polykrikoides*, the culture was not filtered prior to its addition to the appropriate experimental bottles and, therefore, 16s data was not evaluated.

## Discussion

Biotic interactions are likely to have a myriad of direct and indirect influences on the occurrence of harmful algal blooms. This study identified the microbial consortium associated with *C*. *polykrikoides* blooms and revealed that *C*. *polykrikoides* blooms were enriched in dinoflagellates and depleted in diatoms (>5μm fraction) among eukaryotes, and enriched in Rhodobacteraceae and depleted in Flavobacteriaceae, Rhodospirillaceae and the SAR11 clade among prokaryotes. *Amoebophrya* was highly abundant both within and near blooms while lineages of *Geminigera* sp., *Cylindrotheca closterium*, *Leptocylindrus* spp., an uncultured eukaryote from MAST-3J, and *Pirsonia* sp., were unique members of the core eukaryotic microbiomes found inside blooms. In contrast, lineages of *Gonyaulax* sp., *Rhaphoneis* sp. (Bacillariophyceae), two cryptophytes (*Katablepharis* sp., *Leucocryptos* sp.), two Prymnesiales (uncultured eukaryote and OLI16029), two uncultured eukaryotes (Mamiellophyceae and Chrysophyceae), and an uncultured eukaryote from the Syndiniales were only found outside of blooms. Among microbes, two lineages (an unassigned bacterium, and an uncultured bacterium from Rickettsiales) from the >0.2μm fraction, and one (the Gammaproteobacteria, *Halioglobus*) from the >5μm fraction were unique to the core microbiome of blooms. Collectively, these observations provide new insight into the composition and potential function of the eukaryotic and prokaryotic communities associated with and inhibited by *C*. *polykrikoides* bloom.

Similar to prior studies [33,39,49], high-throughput sequencing facilitated the discovery of a number of species that have yet to be described in *C*. *polykrikoides* blooms in the western hemisphere. High-throughput amplicon sequencing provided an enhanced resolution of phytoplankton community diversity, revealing parasitic species (i.e. the dinoflagellates, *Amoebophrya* and *Duboscquella*; the nanoflagellate, *Pirsonia*), picoplankton (i.e. *Synechococcus)*, and species found in low abundance (i.e. *Katablepharis* sp., *Leucocryptos* sp.), many of which have yet to be identified via traditional microscopy within blooms. Consistent with prior studies utilizing microscopy, high-throughput sequencing captured most of the common, larger phytoplankton species known to be present during the summer/fall season within this region ([8–10] Hattenrath-Lehmann personal observation). *Prorocentrum*, a dinoflagellate known to co-occur with *C*. *polykrikoides* blooms in New York [9], however, was detected at comparatively lower relative abundances than expected, perhaps due to a lack of sequences of one of the most common species found in NY, *P*. *gracile*, in the SILVA reference database [39]. For this reason, it has been recommended [33,39] that both microscopy and sequencing be utilized for comprehensive descriptions of microbial communities. The importance of- and need for- assessing the full microbiome, including both prokaryotes and eukaryotes has become increasingly obvious as it has been demonstrated that biotic interactions are better predictors of community structure than abiotic factors [64]. In the future, long-term monitoring of the base of the food chain of coastal ecosystems using a combination of microscopy and high-throughput sequencing would provide a more accurate and comprehensive assessment of the full suite of microbial consortia that are likely to improve over time as informative databases expand and become more accurately annotated [35,65].

While the parasitic dinoflagellate *Amoebophrya* spp. is known to form associations with many other dinoflagellate species [66–68], observations of *Amoebophrya* infections during *C*. *polykrikoides* blooms have never been reported outside of Korean waters [68–71]. Here, using high-throughput sequencing, *Amoebophrya* was detected in all samples analyzed, regardless of region, size fraction, or year. Moreover, *Amoebophrya* sequences were 95–100% identical to a Korean isolate from Namhae (NCBI accession KF791348). *Amoebophrya* accounted for up to 54% of the eukaryotic community and was enriched within bloom communities. While *Gyrodinium spirale* and *Polykrikos* spp. were also present and have been previously described as hosts of the parasite *Amoebophrya* [68], *Amoebophrya* was also dominant when these two potential hosts were present at very low relative abundances (<1%; Fig 2). Therefore, while not observed microscopically, we hypothesize that *C*. *polykrikoides* was the primary host of *Amoebophrya*. Using high-throughput sequencing, *Amoebophrya* was also abundant in another NY embayment, Northport Bay, in association with blooms of *Alexandrium catenella* [39]. Parasitic *Amoebophrya* infections may be more-widespread and important to HAB dynamics than previously assumed and may require the use of molecular methods to clarify their importance [71]. Since 2014, blooms of *C*. *polykrikoides* have become less intense in NY, with blooms achieving a lower maximal biomass, persisting for a shorter period of time, and being less widespread than blooms that occurred from 2006–2013 [8,10] (C. Gobler personal observation). While the role of *Amoebophrya* in this change is unknown, adaptation of *Amoebophrya* and/or other parasitic communities toward the exertion of stronger biological control could account for such a shift.

In a manner similar to several other HABs [39,72–75], *C*. *polykrikoides* can shape planktonic community structure via the secretion of allelopathic compounds [23]. Allelochemicals produced by *C*. *polykrikoides* have the capacity to lyse and inhibit the growth of several species of dinoflagellates, diatoms, cryptophytes, haptophytes, raphidophytes, and pelagophytes in culture as well as natural plankton communities with effects being dependent on the densities of *C*. *polykrikoides* as well as target species [23]. Consistent with these known allelopathic capabilities, *C*. *polykrikoides* dominated both in- and outside- of patches, and phytoplankton community diversity was significantly lower within *C*. *polykrikoides* patches compared to non-patch samples, suggesting species vulnerable to allelochemicals were selectively eliminated. Furthermore, *Chaetoceros* and *Gyrodinium* were more abundant outside of *Cochlodinium* patches, a finding consistent with prior observations [9] and the density dependency of these allelopathic effects [23]. In experiments, the addition of *C*. *polykrikoides* to a natural plankton community resulted in the enrichment of *Amoebophrya*, *Gyrodinium spirale*, *Polykrikos*, several other dinoflagellates, *Chaetoceros*, and *Apedinella*, but the depletion of several other genera including *Thalassiosira*, *Picomonas* and *Micromonas* (S22 Fig). Natural community experiments conducted by Tang and Gobler [23] demonstrated that euglena populations were significantly enhanced with the addition of *C*. *polykrikoides* culture, while *Gyrodinium*, *Scrippsiella*, *Skeletonema*, *Chaetoceros* and *Thalassiosira* all significantly decreased. While disparities among these studies (i.e. *Chaetoceros* and *Gyrodinium*) are likely due to density, species, and strain dependent allelopathic effects [23], it is clear that 18S sequencing was capable of describing allelopathic patterns and can enhance allelopathic studies by revealing rare members of the community that would otherwise not have been captured using microscopic methods.

HABs can also influence prokaryotic communities [36–39,76]. To date, only two studies have assessed the microbiome associated with *C*. *polykrikoides* blooms, both using molecular techniques that differed from each other and from the current study [9,32]. Consistent with what was found using clone library analysis of *C*. *polykrikoides* blooms in South Korea [32], *C*. *polykrikoides* blooms during this study were dominated by bacteria of the Alphaproteobacteria and Flavobacteria lineages. In addition, blooms of *C*. *polykrikoides* in NY were also dominated by cyanobacteria, which had higher relative abundances in the >5μm fraction (up to 22%) and were more abundant than in Korean blooms (<5.4%) [32]. Rhodobacterales had higher relative abundances in patches than outside of patches, a finding similar to Park et al. [32] who reported that Rhodobacterales were more abundant during bloom peaks and had a significant positive correlation with *C*. *polykrikoides* cell densities. Similar to NY, during blooms in Korea Alphaproteobacteria were dominated by the SAR11 cluster, Rhodobacterales and Rhodospirillales, but unlike NY blooms where the Gammaproteobacteria, Alteromonadales and Oceanospirillales were consistently present, these bacteria were rarer in Korea [32]. Also consistent with the present study, using terminal restriction fragment length polymorphism (TRFLP) analysis of 16S rRNA genes, Koch et al. [9] found TRFs consistent with those predicted for Alphaproteobacteria were more abundant in bloom samples. Despite the use of different molecular techniques, there were numerous similarities among the present study, Koch et al. [9] and Park et al. [32]. High-throughput sequencing, however, clearly provided a more in depth analysis of the microbial community than the prior studies.

Recently, two studies [40,41] utilized high-throughput sequencing to describe the prokaryotic community associated with Korean cultures of *C*. *polykrikoides*. While the *C*. *polykrikoides* culture isolated by Park et al. [40] was dominated by *Marivita* sp. (Roseobacter) and *Winogradskyella* sp. (Flavobacteria), the culture isolated by Shin et al. [41] was dominated by *Methylophaga*, *Marinobacter*, *Ponticoccus* and *Jannaschia*. In New York, *Marivita* sp. (<3%), *Winogradskyella* sp. (<17%), *Methylophaga* (<0.5%), *Marinobacter* (<0.5%), and *Jannaschia* (<0.05%) were present among field samples at varying relative abundances, however, *Ponticoccus* was not present in the dataset. Among these genera, *Winogradskyella* sp. had the highest relative abundances and differential abundance analyses demonstrated it was more abundant in non-patch samples and significantly enriched in the >5μm fraction (S4 Fig), with a PCA further confirming a strong association between this bacteria and the >5μm fraction. Despite low relative abundances, *Methylophaga* and *Marinobacter* were found to be significantly more abundant in >0.2μm fraction of both patch and non-patch samples. As with any culture, both of the Korean *C*. *polykrikoides* isolates were cultivated under environmental conditions optimal for growth of this alga (i.e. ideal temperature, light intensity, and saturating nutrient concentrations) and these conditions will likely affect the free-living and epiphytic bacteria associated with the cultures. As such, bacteria found in cultures would be expected to differ from those in the field where environmental conditions will differ and are more dynamic. Accordingly, only a single dominant genus (*Winogradskyella* sp.) from Korean cultures [40] was also found at high relative abundances in NY field samples.

Phytoplankton blooms, in general [77], and HABs such as *Alexandrium* spp., *Pseudonitzschia* sp., *Akashiwo sanguinea*, and *C*. *polykrikoides*, are known to be dominated by members of specific heterotrophic bacterial lineages including Alphaproteobacteria, Gammaproteobacteria, and Flavobacteria [32,36,38,39,78–83]. During this study *C*. *polykrikoides* blooms in NY were also dominated by the classes Flavobacteria and Alphaproteobacteria, while Gammaproteobacteria comprised a relatively smaller portion of microbial communities compared to prior HAB studies. Within those classes, *C*. *polykrikoides* blooms shared a series of microbial similarities with blooms of *A*. *catenella* and *Dinophysis acuminata* reported by Hattenrath-Lehmann and Gobler [39] including dominance of the orders Flavobacteriales, Rhodobacterales, and SAR11. While many of the bacteria from these two studies are members of similar lineages [39], the NS5 marine group (Flavobacteriales), an uncultured bacterium from Rhodobacteracea, *Owenweeksia* spp., *Perlucidbaca* spp. and *Limnobacter* spp were the dominant genera among *Alexandrium*-associated field samples while *Dinophysis*-associated field samples were dominated by ‘unassigned bacteria’ and an uncultured bacterium from Rhodobacteracea. Differential abundance analyses demonstrated that *C*. *polykrikoides* blooms were significantly enriched in an uncultured bacterium from Rhodobacteraceae, the core bacterial microbiome of the >0.2μm fraction of patches was dominated by an uncultured bacterium from the SAR11 clade, while the >5μm size fraction was co-dominated by an uncultured bacterium from Rhodobacteraceae and *Coraliomargarita*. Collectively, the high level of phylogenetic resolution provided by high-throughput sequencing demonstrates that each of these NY HABs was associated with distinct microbiomes.

While the impacts of *C*. *polykrikoides* blooms on eukaryotic community composition and diversity were highly significant, the effects on bacterial communities were somewhat less intense. There were 154 16S OTUs that were unique to the >0.2μm, non-bloom samples, suggesting these 154 OTUs were inhibited by allelochemicals present within blooms. This finding is consistent with the observations of Koch et al. [9] who reported on four 16S T-RFLP fragments found only in non-bloom samples. There were, however, only five OTUs (four in the >0.2μm, one in the >5μm) unique to *C*. *polykrikoides* blooms and diversity analyses did not reveal significant differences between bloom and non-bloom samples for the 16S community. Given these bloom patches move horizontally with currents and tides, it is plausible that the shorter generation times of bacteria make these communities less vulnerable to the effects of *C*. *polykrikoides*, compared to eukaryotes that have significantly slower growth rates [84]. Furthermore, the cell wall of bacteria may make them more resistant to reactive oxygen species and similar allelochemicals released by *C*. *polykrikoides* [85]. Greater differences between bacterial communities may be more likely to manifest themselves over the course of the blooms as described in Park et al. [32] as organic matter inventories build and temperatures change [86]. There were far more significant differences between the size fractions within patch and non-patch samples among bacterial communities (S6–S21 Figs), demonstrating the strong differences in particle associated bacteria vs. free-living bacteria.

Predictive tools (PICRUST2) used to determine functional potential of microbial communities revealed significant differences between size fractions, and size fractions within- and between- regions. For example, on multiple dates, the predicted metagenomic communities associated with bloom patches had significantly higher levels of metabolic pathways associated with the cytochrome c aerobic pathway than non-bloom samples. Beyond its role in mitochondrial respiration and the electron transport chain, cytochrome c can also act as an anti-oxidative enzyme, removing reactive oxygen species (ROS) [87,88]. Given that the toxic effects of *C*. *polykrikoides* are known to emanate from the production of ROS [3,16,85], this finding suggests that bacteria with a greater abundance of cytochrome c may be better suited to co-exist with dense blooms of *C*. *polykrikoides* where ROS-production is presumably high. Still, metagenomic predictive tools should be taken with caution given that functional potential results are generated from a limited database of available genomes.

The roles of the dominant bacteria found associated with *C*. *polykrikoides* blooms can also be surmised from the literature. For example, patch samples were dominated by bacteria such as *Coraliomargarita*, part of the phylum Verrucomicrobia, which preferentially grow on phytoplankton-derived high molecular organic compounds [89] that would likely be readily available in bloom formers such as *C*. *polykrikoides* that excrete extracellular polysaccharides [9,16,90]. Flavobacteria (*Winogradskyella* co-dominate in patches) are known to facilitate macromolecule conversions, converting high molecular compounds to low molecular weight compounds [77], a process that may concurrently support the growth of *C*. *polykrikoides* by providing smaller, labile compounds such as glutamic acid which this alga consumes readily [8]. Other microbial members that co-dominated within *C*. *polykrikoides* patches, such as bacterium from the AEGEAN-169 marine group and the SAR11 clade, are part of the phylum Alphaproteobacteria which are capable of assimilating dissolved organic matter (DOM) including extracellular polymeric substances (EPS) [91] which are produced in copious amounts by *C*. *polykrikoides* [16,90]. Beyond dominant microbes, it has been demonstrated that if a rarer microbe is responsible for a key metabolic process that other microbes do not carry out, it can have an enormous ecosystem impact [92] and the discovery of the metabolic repertoire of rare microbes will likely continue to advance in parallel with high-throughput sequencing efforts. *C*. *polykrikoides* has an obligate requirement for B-vitamins [18] and it has been demonstrated that the availability of both, B_1_ and B_12_ can shape bloom dynamics [9,93]. Vitamin concentrations and turnover inside bloom patches of *C*. *polykrikoides* are elevated, a fact not only attributed to the auxotrophic phytoplankton but also to the bacterial consortium residing within the patches [9]. While the relative abundance of potential B_12_ producing-bacteria belonging to the order Rhodobacterales (class Alphaproteobacteria) [94,95] was significantly enriched in patch vs non-patch samples, the former has been shown to harbor 10-fold higher heterotrophic bacterial densities, likely leading to the observed higher vitamin concentrations found in Koch et al. [9]. Interestingly, Rhodobacterales was also more abundant in the >5μm fraction of patch samples, suggesting a potentially endosymbiotic association between these potential vitamin producers and auxotrophic *C*. *polykrikoides*.

*Synechococcus* was part of the core microbiome but was significantly enriched in the >5μm fraction of both patch and non-patch samples, despite it being a 1 μm cell. *C*. *polykrikoides* can feed on *Synechococcus* [20,96,97]. Field samples revealed that the >5μm fraction of patches (1–7%) had slightly lower relative abundances of *Synechococcus* than the same size fraction in non-patch samples (1–13%; Fig 6). This finding is consistent with Koch et al. [9] who found that *Synechococcus* densities were significantly lower in patches of *C*. *polykrikoides* and could indicate *C*. *polykrikoides* is capable of consuming *Synechococcus* and/or inhibiting this population via allelochemicals. Collectively, this suggests that these cyanobacteria may influence the nutrition of *C*. *polykrikoides* and potentially the C and N cycles of the surrounding microbiome.

In summary, this study revealed the profound effects of *C*. *polykrikoides* blooms on prokaryotic and eukaryotic plankton communities. The relative abundance of dinoflagellates in general, and *C*. *polykrikoides* and *Amoebophrya* in particular, were enriched in bloom samples and the experimental enrichment of *C*. *polykrikoides* led to a significant increase in the relative abundance of eight genera of dinoflagellates but a significant decline in other eukaryotic plankton. Patch and non-patch eukaryotic community composition were significantly different and patch communities harbored significantly lower diversity than non-patch samples, with more than 30 unique OTUs found within non-patch samples, suggesting blooms allelopathically inhibited these OTUs. While differential abundance analyses demonstrated that the Rhodobacterales were significantly enriched in *C*. *polykrikoides* patches and that there were more than 150 unique OTUs in non-bloom samples, the overall impact of these HABs on bacterial community composition and diversity was less intense compared to the impacts on eukaryotic communities.

## Supporting information

S1 Table QIIME outputs for the sequencing of the V7/V8 region of the 18S rRNA gene, including split libraries output (demultiplexed reads), total UCLUST assigned reads and the total number of algal assigned reads for time series (patch and non-patch), and experimental samples (initial, control and Cochlodinium addition) and size fractions (>0.2 and >5μm). % algal reads = (total number of algal assigned reads divided by the total number of UCLUST assigned reads) x 100.
(PDF)

S2 Table QIIME outputs for the sequencing of the V4 variable region of the 16S rRNA gene, including split libraries output (demultiplexed reads), total UCLUST assigned reads and the total number of chloroplast and mitochondria assigned reads for time series (patch and non-patch) samples and size fractions (>0.2 and >5μm). (PDF)

S3 Table Alpha diversity metrics for algal OTUs in field samples for region (patch vs non-patch (NP)), size fraction (>0.2 vs >5μm) and year (2011, 2012 and 2013). **p<0.001 and *p<0.01. SD = standard deviation.
(PDF)

S4 Table Alpha diversity metrics for bacterial OTUs in field samples for region (patch vs non- patch (NP)), size fraction (>0.2 vs >5μm) and year (2011, 2012 and 2013). **p<0.001 and *p<0.01. SD = standard deviation.
(PDF)

S1 Fig Differentially abundant 18S lineages among representative classes (see Fig 2) in patch and non-patch samples for A) >0.2μm and B) >5μm size fractioned samples. Negative log2 fold changes represent lineages enriched in non-patch samples, while positive log2 fold changes represent lineages enriched in patch samples. Significant differential abundances (alpha <0.05) are indicated by red circles.
(PDF)

S2 Fig Genus level differentially abundant 18S lineages among patch and non-patch samples for A) >0.2μm and B) >5μm size fractioned samples. Negative log2 fold changes represent lineages enriched in non-patch samples, while positive log2 fold changes represent lineages enriched in patch samples. Lineages that are part of the patch (+) and non-patch (-) core microbiomes are in bold, and italicized if unique to a core microbiome. Only significant differential abundances (alpha <0.05) are shown. Taxa are colored by order.
(PDF)

S3 Fig Differentially abundant 16S lineages among representative orders (see Fig 5) in A) patch >0.2μm (-) vs patch >5μm (+), B) non-patch >0.2μm (-) vs non-patch >5μm (+), C) non-patch >0.2μm (-) vs patch >0.2μm (+), D) non-patch >5μm (-) vs patch >5μm (+). Negative (-) and positive (+) log2 fold changes represent lineages enriched as above. Significant differential abundances (alpha <0.05) are indicated by red circles, while black circles indicate non-significant values.
(PDF)

S4 Fig Differentially abundant 16S lineages among representative genera (see Fig 6) in A) patch >0.2μm (-) vs patch >5μm (+), B) non-patch >0.2μm (-) vs non-patch >5μm (+), C) non-patch >0.2μm (-) vs patch >0.2μm (+), D) non-patch >5μm (-) vs patch >5μm (+). Negative (-) and positive (+) log2 fold changes represent lineages enriched as above. Significant differential abundances (alpha <0.05) are indicated by red circles, while black circles indicate non-significant values.
(PDF)

S5 Fig Genus level differentially abundant 16S lineages among patch and non-patch samples for A) >0.2μm and B) >5μm size fractioned samples. Negative log2 fold changes represent lineages enriched in non-patch samples, while positive log2 fold changes represent lineages enriched in patch samples. Lineages that are part of the patch (+) and non-patch (-) core microbiomes are in bold, and italicized if unique to a core microbiome. Only significant differential abundances (alpha <0.05) are shown. Taxa are colored by order.
(PDF)

S6 Fig Differentially abundant 16S lineages among the Patch >0.2μm and Patch >5μm size fraction samples for A) Acidobacteria and B) Actinobacteria. Negative log2 fold changes represent lineages enriched in >0.2μm samples, while positive log2 fold changes represent lineages enriched in >5μm samples. Lineages that are part of the >5μm fraction (+) and >0.2μm fraction (-) core microbiomes are in bold, and italicized if unique to a core microbiome. Only significant differential abundances (alpha <0.05) are shown. Data is grouped by phyla (taxonomy) and colored by order. Unknown orders are listed as next lowest known taxonomy and indicated with *.
(PDF)

S7 Fig Differentially abundant 16S lineages among the Patch >0.2μm and Patch >5μm size fraction samples for Bacteriodetes. Negative log2 fold changes represent lineages enriched in >0.2μm samples, while positive log2 fold changes represent lineages enriched in >5μm samples. Lineages that are part of the >5μm fraction (+) and >0.2μm fraction (-) core microbiomes are in bold, and italicized if unique to a core microbiome. Only significant differential abundances (alpha <0.05) are shown. Data is grouped by phyla (taxonomy) and colored by order. Unknown orders are listed as next lowest known taxonomy and indicated with *.
(PDF)

S8 Fig Differentially abundant 16S lineages among the Patch >0.2μm and Patch >5μm size fraction samples for Alphaproteobacteria. Negative log2 fold changes represent lineages enriched in >0.2μm samples, while positive log2 fold changes represent lineages enriched in >5μm samples. Lineages that are part of the >5μm fraction (+) and >0.2μm fraction (-) core microbiomes are in bold, and italicized if unique to a core microbiome. Only significant differential abundances (alpha <0.05) are shown. Data is grouped by phyla (taxonomy) and colored by order. Unknown orders are listed as next lowest known taxonomy and indicated with *.
(PDF)

S9 Fig Differentially abundant 16S lineages among the Patch >0.2μm and Patch >5μm size fraction samples for A) Betaproteobacteria and B) Deltaproteobacteria. Negative log2 fold changes represent lineages enriched in >0.2μm samples, while positive log2 fold changes represent lineages enriched in >5μm samples. Lineages that are part of the >5μm fraction (+) and >0.2μm fraction (-) core microbiomes are in bold, and italicized if unique to a core microbiome. Only significant differential abundances (alpha <0.05) are shown. Data is grouped by phyla (taxonomy) and colored by order. Unknown orders are listed as next lowest known taxonomy and indicated with *.
(PDF)

S10 Fig Differentially abundant 16S lineages among the Patch >0.2μm and Patch >5μm size fraction samples for Gammaproteobacteria. Negative log2 fold changes represent lineages enriched in >0.2μm samples, while positive log2 fold changes represent lineages enriched in >5μm samples. Lineages that are part of the >5μm fraction (+) and >0.2μm fraction (-) core microbiomes are in bold, and italicized if unique to a core microbiome. Only significant differential abundances (alpha <0.05) are shown. Data is grouped by phyla (taxonomy) and colored by order. Unknown orders are listed as next lowest known taxonomy and indicated with *.
(PDF)

S11 Fig Differentially abundant 16S lineages among the Patch >0.2μm and Patch >5μm size fraction samples for A) other Proteobacteria and B) Planctomycetes. Negative log2 fold changes represent lineages enriched in >0.2μm samples, while positive log2 fold changes represent lineages enriched in >5μm samples. Lineages that are part of the >5μm fraction (+) and >0.2μm fraction (-) core microbiomes are in bold, and italicized if unique to a core microbiome. Only significant differential abundances (alpha <0.05) are shown. Data is grouped by phyla (taxonomy) and colored by order. Unknown orders are listed as next lowest known taxonomy and indicated with *.
(PDF)

S12 Fig Differentially abundant 16S lineages among the Patch >0.2μm and Patch >5μm size fraction samples for A) SAR, B) Lentisphaerae and C) Verrucomicrobia. Negative log2 fold changes represent lineages enriched in >0.2μm samples, while positive log2 fold changes represent lineages enriched in >5μm samples. Lineages that are part of the >5μm fraction (+) and >0.2μm fraction (-) core microbiomes are in bold, and italicized if unique to a core microbiome. Only significant differential abundances (alpha <0.05) are shown. Data is grouped by phyla (taxonomy) and colored by order. Unknown orders are listed as next lowest known taxonomy and indicated with *.
(PDF)

S13 Fig Differentially abundant 16S lineages among the Patch >0.2μm and Patch >5μm size fraction samples for A) Other and B) Chlorobi and Chloroflexi. Negative log2 fold changes represent lineages enriched in >0.2μm samples, while positive log2 fold changes represent lineages enriched in >5μm samples. Lineages that are part of the >5μm fraction (+) and >0.2μm fraction (-) core microbiomes are in bold, and italicized if unique to a core microbiome. Only significant differential abundances (alpha <0.05) are shown. Data is grouped by phyla (taxonomy) and colored by order. Unknown orders are listed as next lowest known taxonomy and indicated with *.
(PDF)

S14 Fig Differentially abundant 16S lineages among the Non-patch >0.2μm and Non-patch >5μm size fraction samples for A) Acidobacteria and B) Actinobacteria. Negative log2 fold changes represent lineages enriched in >0.2μm samples, while positive log2 fold changes represent lineages enriched in >5μm samples. Lineages that are part of the >5μm fraction (+) and >0.2μm fraction (-) core microbiomes are in bold, and italicized if unique to a core microbiome. Only significant differential abundances (alpha <0.05) are shown. Data is grouped by phyla (taxonomy) and colored by order. Unknown orders are listed as next lowest known taxonomy and indicated with *.
(PDF)

S15 Fig Differentially abundant 16S lineages among the Non-patch >0.2μm and Non-patch >5μm size fraction samples for Bacteriodetes. Negative log2 fold changes represent lineages enriched in >0.2μm samples, while positive log2 fold changes represent lineages enriched in >5μm samples. Lineages that are part of the >5μm fraction (+) and >0.2μm fraction (-) core microbiomes are in bold, and italicized if unique to a core microbiome. Only significant differential abundances (alpha <0.05) are shown. Data is grouped by phyla (taxonomy) and colored by order. Unknown orders are listed as next lowest known taxonomy and indicated with *.
(PDF)

S16 Fig Differentially abundant 16S lineages among the Non-patch >0.2μm and Non-patch >5μm size fraction samples for Alphaproteobacteria. Negative log2 fold changes represent lineages enriched in >0.2μm samples, while positive log2 fold changes represent lineages enriched in >5μm samples. Lineages that are part of the >5μm fraction (+) and >0.2μm fraction (-) core microbiomes are in bold, and italicized if unique to a core microbiome. Only significant differential abundances (alpha <0.05) are shown. Data is grouped by phyla (taxonomy) and colored by order. Unknown orders are listed as next lowest known taxonomy and indicated with *.
(PDF)

S17 Fig Differentially abundant 16S lineages among the Non-patch >0.2μm and Non-patch >5μm size fraction samples for A) Deltaproteobacteria and B) Betaproteobacteria. Negative log2 fold changes represent lineages enriched in >0.2μm samples, while positive log2 fold changes represent lineages enriched in >5μm samples. Lineages that are part of the >5μm fraction (+) and >0.2μm fraction (-) core microbiomes are in bold, and italicized if unique to a core microbiome. Only significant differential abundances (alpha <0.05) are shown. Data is grouped by phyla (taxonomy) and colored by order. Unknown orders are listed as next lowest known taxonomy and indicated with *.
(PDF)

S18 Fig Differentially abundant 16S lineages among the Non-patch >0.2μm and Non-patch >5μm size fraction samples for Gammaproteobacteria. Negative log2 fold changes represent lineages enriched in >0.2μm samples, while positive log2 fold changes represent lineages enriched in >5μm samples. Lineages that are part of the >5μm fraction (+) and >0.2μm fraction (-) core microbiomes are in bold, and italicized if unique to a core microbiome. Only significant differential abundances (alpha <0.05) are shown. Data is grouped by phyla (taxonomy) and colored by order. Unknown orders are listed as next lowest known taxonomy and indicated with *.
(PDF)

S19 Fig Differentially abundant 16S lineages among the Non-patch >0.2μm and Non-patch >5μm size fraction samples for A) other Proteobacteria and B) Planctomycetes. Negative log2 fold changes represent lineages enriched in >0.2μm samples, while positive log2 fold changes represent lineages enriched in >5μm samples. Lineages that are part of the >5μm fraction (+) and >0.2μm fraction (-) core microbiomes are in bold, and italicized if unique to a core microbiome. Only significant differential abundances (alpha <0.05) are shown. Data is grouped by phyla (taxonomy) and colored by order. Unknown orders are listed as next lowest known taxonomy and indicated with *.
(PDF)

S20 Fig Differentially abundant 16S lineages among the Non-patch >0.2μm and Non-patch >5μm size fraction samples for A) Other and B) Chlorobi and Chloroflexi. Negative log2 fold changes represent lineages enriched in >0.2μm samples, while positive log2 fold changes represent lineages enriched in >5μm samples. Lineages that are part of the >5μm fraction (+) and >0.2μm fraction (-) core microbiomes are in bold, and italicized if unique to a core microbiome. Only significant differential abundances (alpha <0.05) are shown. Data is grouped by phyla (taxonomy) and colored by order. Unknown orders are listed as next lowest known taxonomy and indicated with *.
(PDF)

S21 Fig Differentially abundant 16S lineages among the Non-patch >0.2μm and Non-patch >5μm size fraction samples for A) SAR and B) Verrucomicrobia. Negative log2 fold changes represent lineages enriched in >0.2μm samples, while positive log2 fold changes represent lineages enriched in >5μm samples. Lineages that are part of the >5μm fraction (+) and >0.2μm fraction (-) core microbiomes are in bold, and italicized if unique to a core microbiome. Only significant differential abundances (alpha <0.05) are shown. Data is grouped by phyla (taxonomy) and colored by order. Unknown orders are listed as next lowest known taxonomy and indicated with *.
(PDF)

S22 Fig Differentially abundant 18S lineages among the control and treatments of experimental samples. Negative log2 fold changes represent lineages enriched in control samples, while positive log2 fold changes represent lineages enriched in treatment samples.
(PDF)
