# Supplementary material for: Prokaryotic and eukaryotic microbiomes associated with blooms of the ichthyotoxic dinoflagellate Cochlodinium (Margalefidinium) polykrikoides in New York, USA, estuaries
Source: PLoS One. 2019 Nov 7;14(11):e0223067. doi: 10.1371/journal.pone.0223067 (PMC6837389; doi:10.1371/journal.pone.0223067)
Supplement: S1 Table — % algal reads = (total number of algal assigned reads divided by the total number of UCLUST assigned reads) x 100. (PDF) [file pone.0223067.s001.pdf]

S1 Table.

Date	Location	size fraction	split libraries output	UCLUST assigned total reads	UCLUST assigned algal reads	% algal reads
**<u>Time series</u>**						
	Unassigned		568880	524286	420138	80
8/26/2011	Patch	0.2	81500	76863	66416	86
8/26/2011	Patch	5	77909	74003	61592	83
8/26/2011	Non-Patch	0.2	85612	79258	62724	79
8/26/2011	Non-Patch	5	93204	89297	67109	75
8/30/2011	Patch	0.2	113276	109541	106605	97
8/30/2011	Patch	5	119183	115047	104083	90
8/30/2011	Non-Patch	0.2	97483	92111	67437	73
8/30/2011	Non-Patch	5	98697	93680	47072	50
8/28/2012	Patch	0.2	97773	94288	86797	92
8/28/2012	Patch	5	71719	68589	55494	81
8/28/2012	Non-Patch	0.2	89837	83226	62930	76
8/28/2012	Non-Patch	5	91895	86168	61316	71
9/6/2012	Patch	0.2	143499	138587	131367	95
9/6/2012	Patch	5	91065	87943	79150	90
9/6/2012	Non-Patch	0.2	105526	98862	83831	85
9/6/2012	Non-Patch	5	94009	88747	68385	77
8/30/2013	Patch	0.2	115747	112328	91781	82
8/30/2013	Patch	5	130350	126512	103975	82
8/30/2013	Non-Patch	0.2	91934	86706	48129	56
8/30/2013	Non-Patch	5	113736	109048	26622	24
10/2/2013	Patch	0.2	109190	105849	100384	95
10/2/2013	Patch	5	122061	118696	102247	86
10/2/2013	Non-Patch	0.2	120093	112967	58084	51
10/2/2013	Non-Patch	5	98738	94253	61110	65
**<u>Experiment</u>**						
10/29/2014	control #1	0.2	79252	73668	55838	76
10/29/2014	control #2	0.2	76502	70608	56980	81
10/29/2014	control #3	0.2	68295	62796	48731	78
10/29/2014	addition #1	0.2	88559	85723	82569	96
10/29/2014	addition #2	0.2	92862	89823	85578	95
10/29/2014	addition #3	0.2	98707	95612	92265	96
		Total	3527093	3345085	2646739	79
