# Supplementary material for: Prokaryotic and eukaryotic microbiomes associated with blooms of the ichthyotoxic dinoflagellate Cochlodinium (Margalefidinium) polykrikoides in New York, USA, estuaries
Source: PLoS One. 2019 Nov 7;14(11):e0223067. doi: 10.1371/journal.pone.0223067 (PMC6837389; doi:10.1371/journal.pone.0223067)
Supplement: S2 Table — (PDF) [file pone.0223067.s002.pdf]

S2 Table.

Date	Location	size fraction	split libraries output	UCLUST output	# of reads assigned as chloroplasts	# of reads assigned as mitochondria
**<u>Time series</u>**						
	Unassigned		652966	642519	153816	714
8/26/2011	Patch	0.2	172119	170410	15328	81
8/26/2011	Patch	5	43094	42178	19708	23
8/26/2011	Non-Patch	0.2	191064	189490	18437	78
8/26/2011	Non-Patch	5	85387	84464	40464	54
8/30/2011	Patch	0.2	57336	56409	12718	38
8/30/2011	Patch	5	36709	36134	28867	8
8/30/2011	Non-Patch	0.2	168121	165774	37141	72
8/30/2011	Non-Patch	5	141834	140577	107068	47
8/28/2012	Patch	0.2	56088	55372	5083	31
8/28/2012	Patch	5	91937	91126	19899	112
8/28/2012	Non-Patch	0.2	237883	234397	29497	96
8/28/2012	Non Patch	5	156541	154454	59786	83
9/6/2012	Patch	0.2	162222	160867	24216	1187
9/6/2012	Patch	5	31761	31388	16585	508
9/6/2012	Non-Patch	0.2	259628	257585	24648	495
9/6/2012	Non-Patch	5	194326	191928	121061	393
8/30/2013	Patch	0.2	173522	172566	4071	81
8/30/2013	Patch	5	57210	56307	6706	35
8/30/2013	Non-Patch	0.2	89494	89014	2129	31
8/30/2013	Non-Patch	5	130789	128501	18193	57
10/2/2013	Patch	0.2	91458	90373	6970	108
10/2/2013	Patch	5	39568	38355	21485	39
10/2/2013	Non-Patch	0.2	217055	214851	11936	131
10/2/2013	Non-Patch	5	123300	122305	64358	68
		Total	3661412	3617344	870170	4570
