# Supplementary material for: Prokaryotic and eukaryotic microbiomes associated with blooms of the ichthyotoxic dinoflagellate Cochlodinium (Margalefidinium) polykrikoides in New York, USA, estuaries
Source: PLoS One. 2019 Nov 7;14(11):e0223067. doi: 10.1371/journal.pone.0223067 (PMC6837389; doi:10.1371/journal.pone.0223067)
Supplement: S3 Table — **p<0.001 and *p<0.01. SD = standard deviation. (PDF) [file pone.0223067.s003.pdf]

S3 Table.

Test	Group1	Group2	Group1 mean (SD)	Group2 mean (SD)
Chao1	NP	Patch	5839 (945) **	3181 (1004)
	2011	2012	4854 (1581)	4905 (1469)
	2013	2012	3771 (1636)	4905 (1469)
	2011	2013	4854 (1581)	3771 (1636)
	0.2	5	4990 (1866)	4030 (1222)
Shannon	NP	Patch	6.4 (0.7) **	3.8 (1.6)
	2011	2012	5.5 (1.4)	5.7 (1.3)
	2013	2012	4.1 (2.1)	5.7 (1.3)
	2011	2013	5.5 (1.4)	4.1 (2.1)
	0.2	5	5.3 (1.9)	4.9 (1.7)
Simpson	NP	Patch	0.9 (0.1) *	0.7 (0.2)
	2011	2012	0.9 (0.1)	0.9 (0.1)
	2013	2012	0.6 (0.3)	0.9 (0.1)
	2011	2013	0.9 (0.1)	0.6 (0.3)
	0.2	5	0.8 (0.2)	0.8 (0.2)
