# Supplementary material for: Prokaryotic and eukaryotic microbiomes associated with blooms of the ichthyotoxic dinoflagellate Cochlodinium (Margalefidinium) polykrikoides in New York, USA, estuaries
Source: PLoS One. 2019 Nov 7;14(11):e0223067. doi: 10.1371/journal.pone.0223067 (PMC6837389; doi:10.1371/journal.pone.0223067)
Supplement: S4 Table — **p<0.001 and *p<0.01. SD = standard deviation. (PDF) [file pone.0223067.s004.pdf]

S4 Table.

Test	Group1	Group2	Group1 mean (SD)	Group2 mean (SD)
Chao1	NP	Patch	1547 (578)	1332 (471)
	2011	2012	1479 (426)	1803 (570)
	2013	2012	1037 (258)	1803 (570)*
	2011	2013	1479 (426)	1037 (258)
	0.2	5	1114 (201)	1765 (571)*
Shannon	NP	Patch	6.6 (1.0)	6.6 (0.9)
	2011	2012	6.7 (0.9)	7.1 (0.9)
	2013	2012	6.0 (0.7)	7.1 (0.9)
	2011	2013	6.7 (0.9)	6.0 (0.7)
	0.2	5	5.8 (0.4)	7.4 (0.7)**
Simpson	NP	Patch	0.9 (0.03)	0.9 (0.03)
	2011	2012	0.9 (0.03)	0.9 (0.02)
	2013	2012	0.9 (0.03)	0.9 (0.02)
	2011	2013	0.9 (0.03)	0.9 (0.03)
	0.2	5	0.92 (0.02)	0.98 (0.01)**
