# Supplementary figures and images for: Prokaryotic and eukaryotic microbiomes associated with blooms of the ichthyotoxic dinoflagellate Cochlodinium (Margalefidinium) polykrikoides in New York, USA, estuaries
Source: PLoS One. 2019 Nov 7;14(11):e0223067. doi: 10.1371/journal.pone.0223067 (PMC6837389; doi:10.1371/journal.pone.0223067)

S1 Fig.

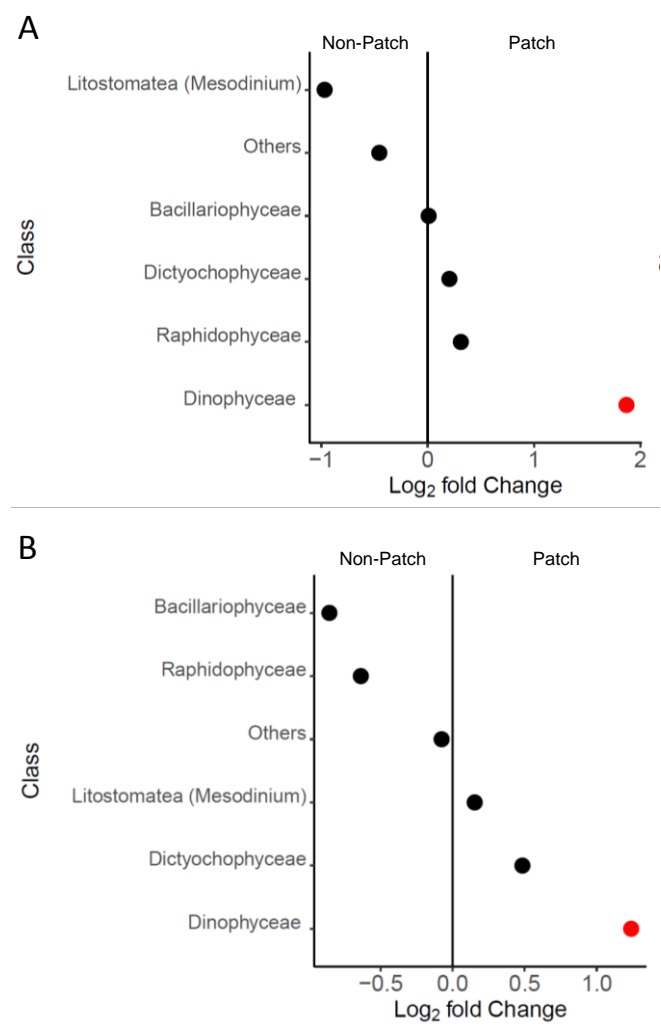

Supplement: S1 Fig — Differentially abundant 18S lineages among representative classes (see Fig 2) in patch and non-patch samples for A) >0.2μm and B) >5μm size fractioned samples. Negative log2 fold changes represent lineages enriched in non-patch samples, while positive log2 fold changes represent lineages enriched in patch samples. Significant differential abundances (alpha <0.05) are indicated by red circles. (PDF) [file pone.0223067.s005.pdf]

S2 Fig.

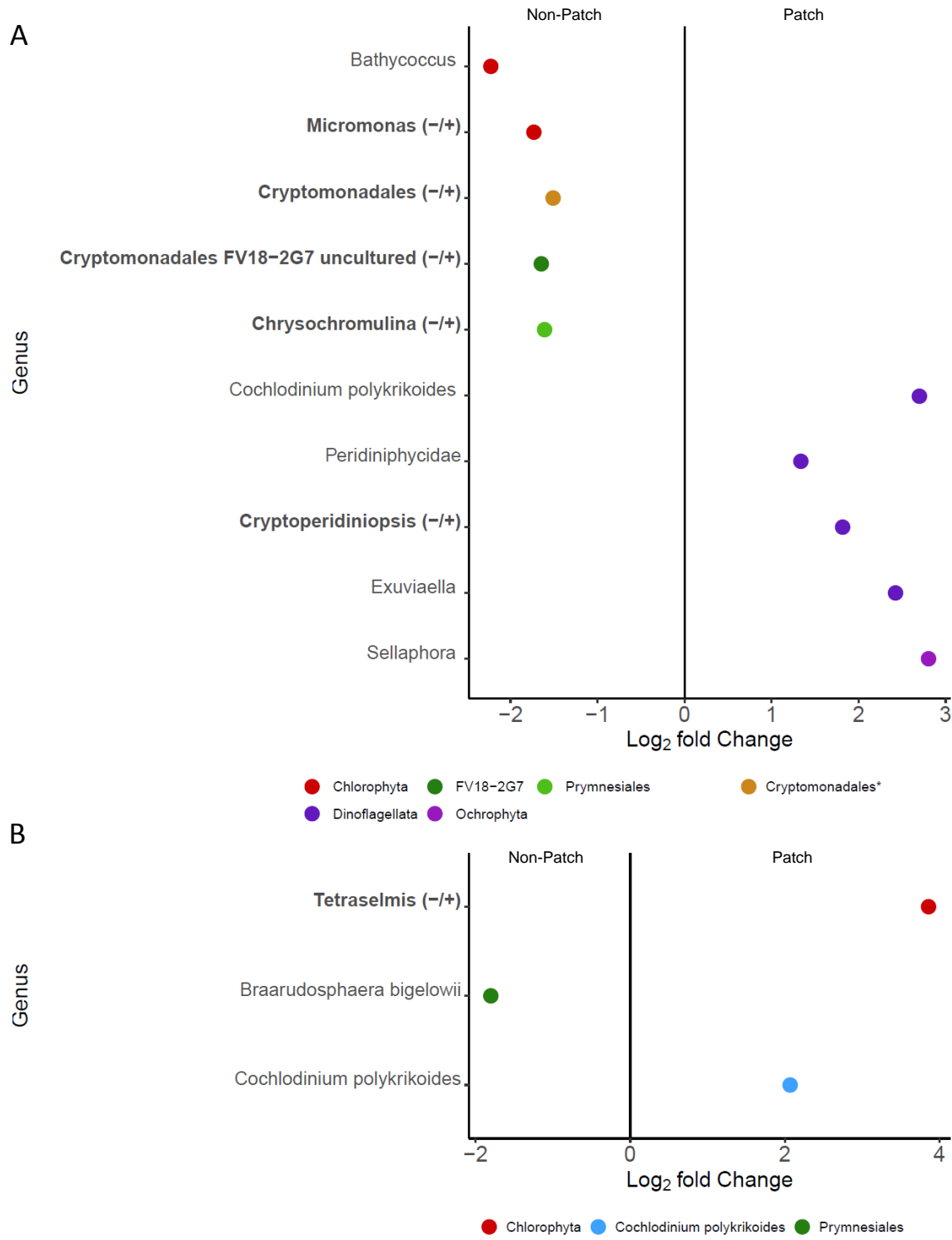

Supplement: S2 Fig — Genus level differentially abundant 18S lineages among patch and non-patch samples for A) >0.2μm and B) >5μm size fractioned samples. Negative log2 fold changes represent lineages enriched in non-patch samples, while positive log2 fold changes represent lineages enriched in patch samples. Lineages that are part of the patch (+) and non-patch (-) core microbiomes are in bold, and italicized if unique to a core microbiome. Only significant differential abundances (alpha <0.05) are shown. Taxa are colored by order. (PDF) [file pone.0223067.s006.pdf]

S3 Fig.

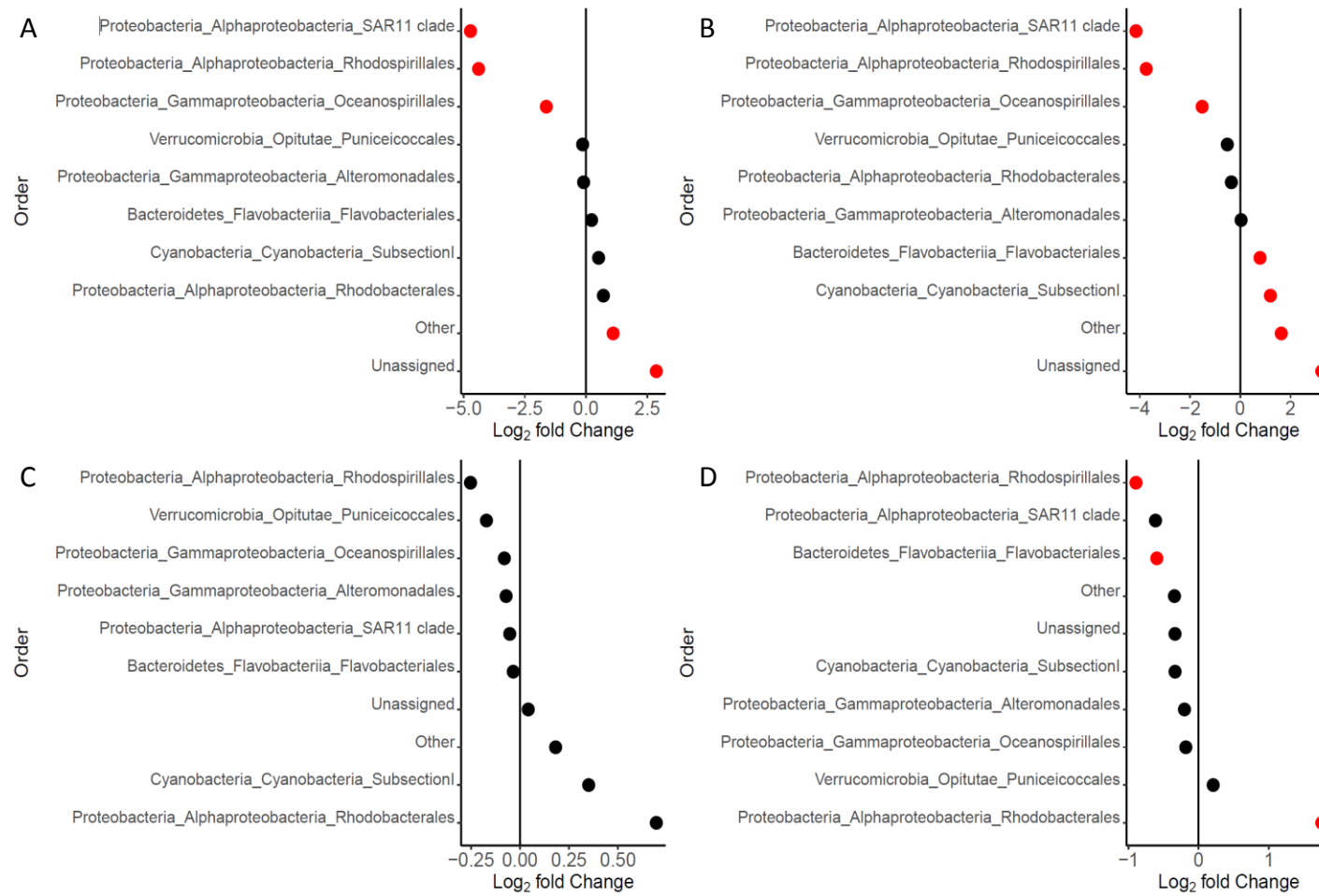

Supplement: S3 Fig — Differentially abundant 16S lineages among representative orders (see Fig 5) in A) patch >0.2μm (-) vs patch >5μm (+), B) non-patch >0.2μm (-) vs non-patch >5μm (+), C) non-patch >0.2μm (-) vs patch >0.2μm (+), D) non-patch >5μm (-) vs patch >5μm (+). Negative (-) and positive (+) log2 fold changes represent lineages enriched as above. Significant differential abundances (alpha <0.05) are indicated by red circles, while black circles indicate non-significant values. (PDF) [file pone.0223067.s007.pdf]

S4 Fig.

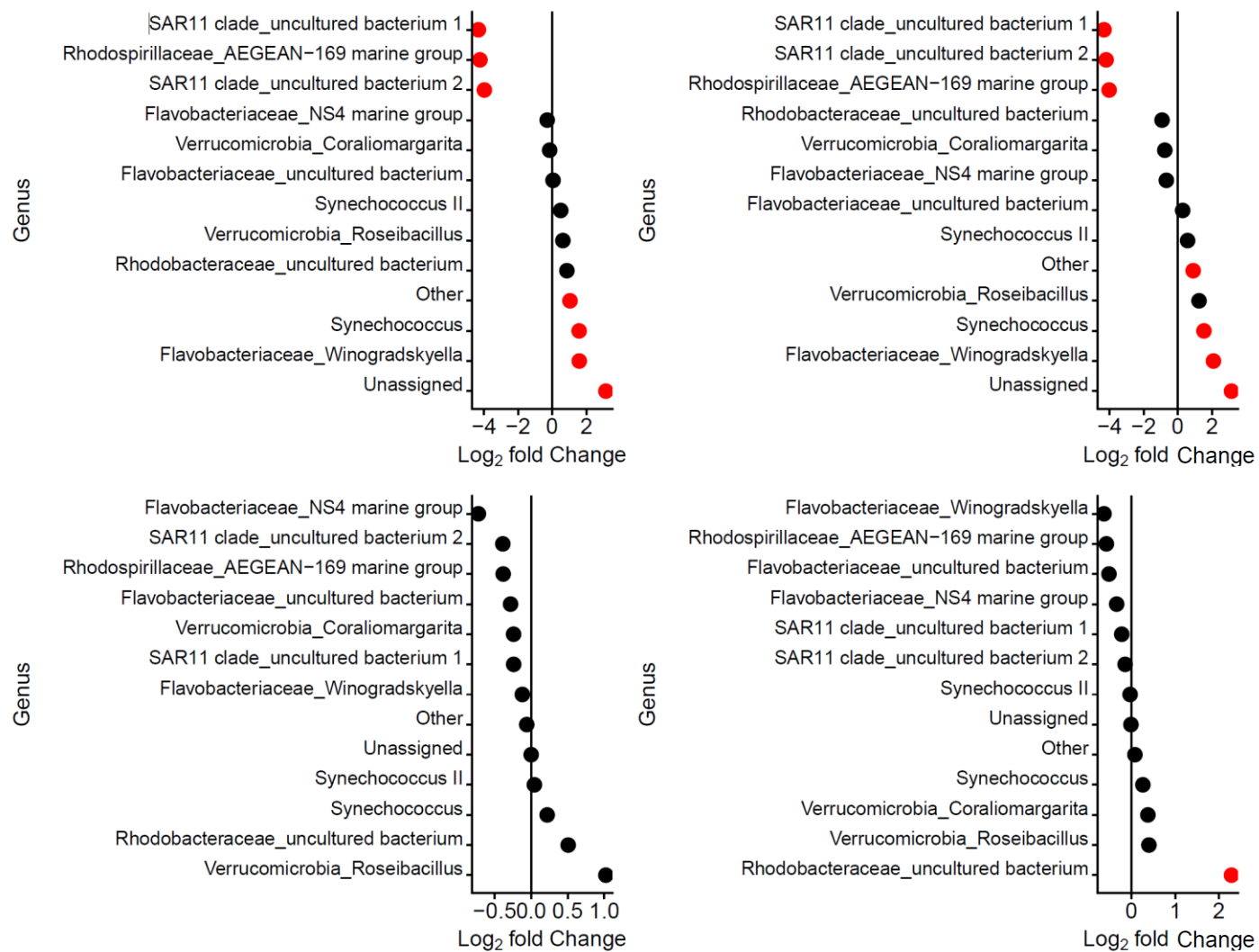

Supplement: S4 Fig — Differentially abundant 16S lineages among representative genera (see Fig 6) in A) patch >0.2μm (-) vs patch >5μm (+), B) non-patch >0.2μm (-) vs non-patch >5μm (+), C) non-patch >0.2μm (-) vs patch >0.2μm (+), D) non-patch >5μm (-) vs patch >5μm (+). Negative (-) and positive (+) log2 fold changes represent lineages enriched as above. Significant differential abundances (alpha <0.05) are indicated by red circles, while black circles indicate non-significant values. (PDF) [file pone.0223067.s008.pdf]

S5 Fig.

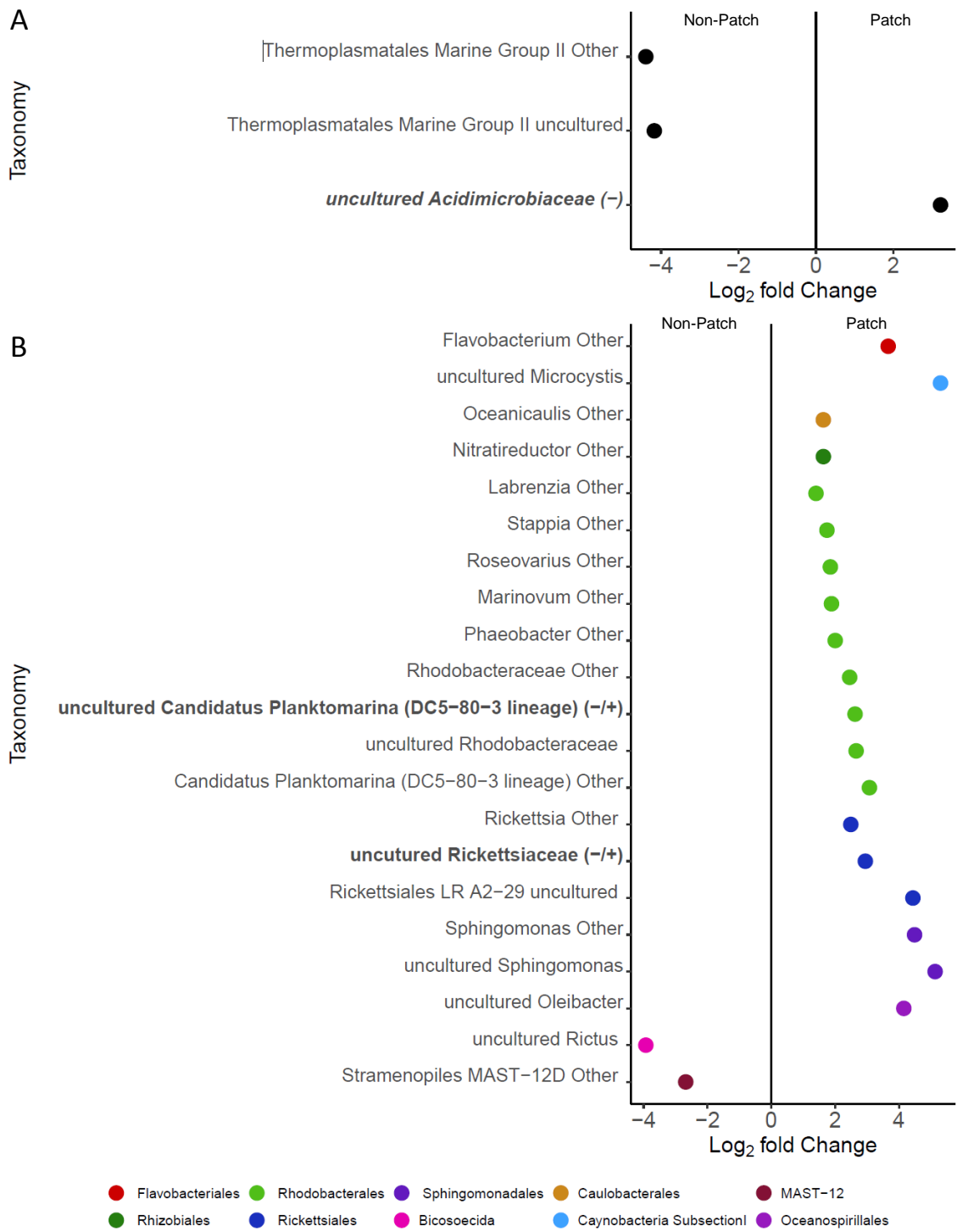

Supplement: S5 Fig — Genus level differentially abundant 16S lineages among patch and non-patch samples for A) >0.2μm and B) >5μm size fractioned samples. Negative log2 fold changes represent lineages enriched in non-patch samples, while positive log2 fold changes represent lineages enriched in patch samples. Lineages that are part of the patch (+) and non-patch (-) core microbiomes are in bold, and italicized if unique to a core microbiome. Only significant differential abundances (alpha <0.05) are shown. Taxa are colored by order. (PDF) [file pone.0223067.s009.pdf]

S6 Fig.

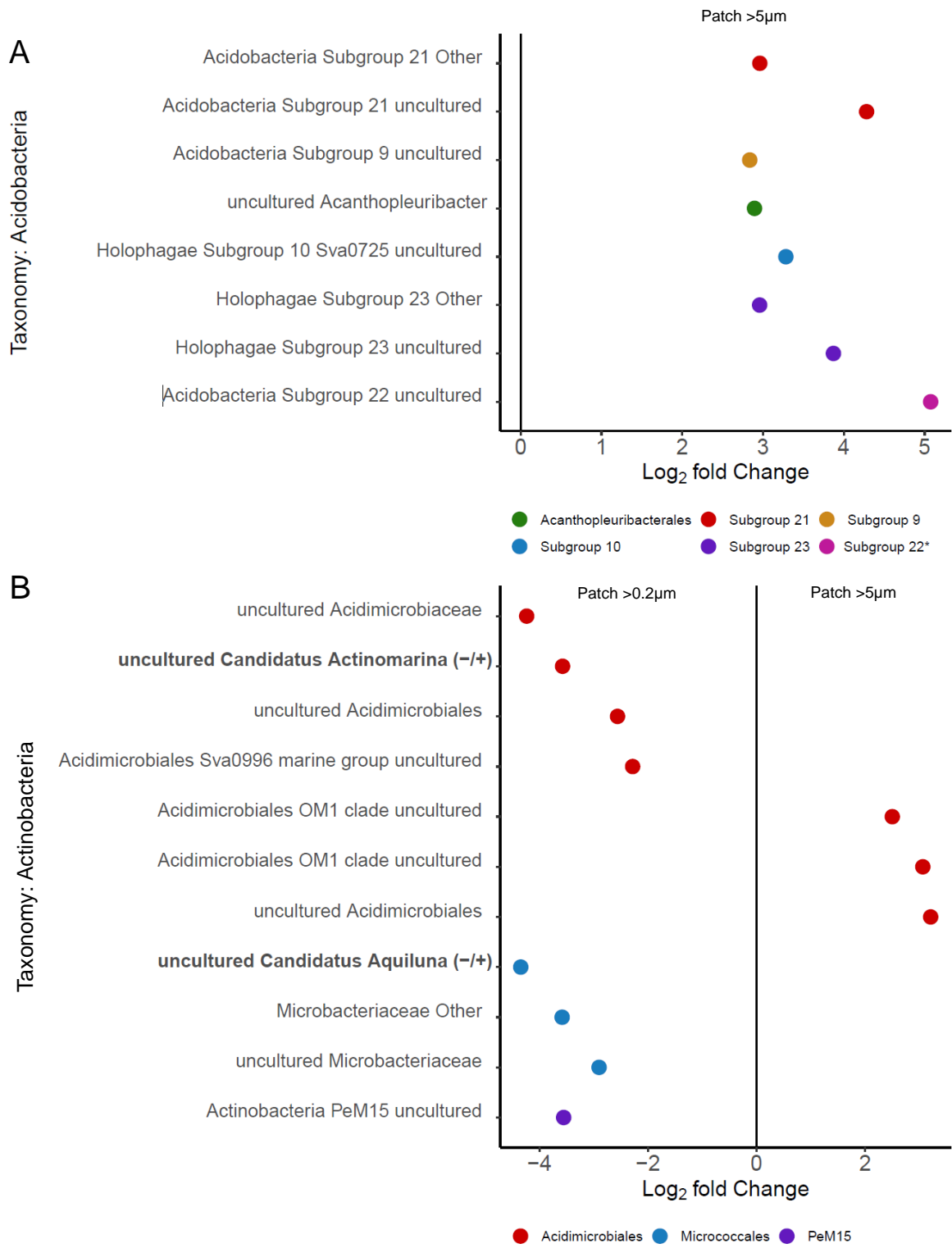

Supplement: S6 Fig — Differentially abundant 16S lineages among the Patch >0.2μm and Patch >5μm size fraction samples for A) Acidobacteria and B) Actinobacteria. Negative log2 fold changes represent lineages enriched in >0.2μm samples, while positive log2 fold changes represent lineages enriched in >5μm samples. Lineages that are part of the >5μm fraction (+) and >0.2μm fraction (-) core microbiomes are in bold, and italicized if unique to a core microbiome. Only significant differential abundances (alpha <0.05) are shown. Data is grouped by phyla (taxonomy) and colored by order. Unknown orders are listed as next lowest known taxonomy and indicated with *. (PDF) [file pone.0223067.s010.pdf]

S7 Fig.

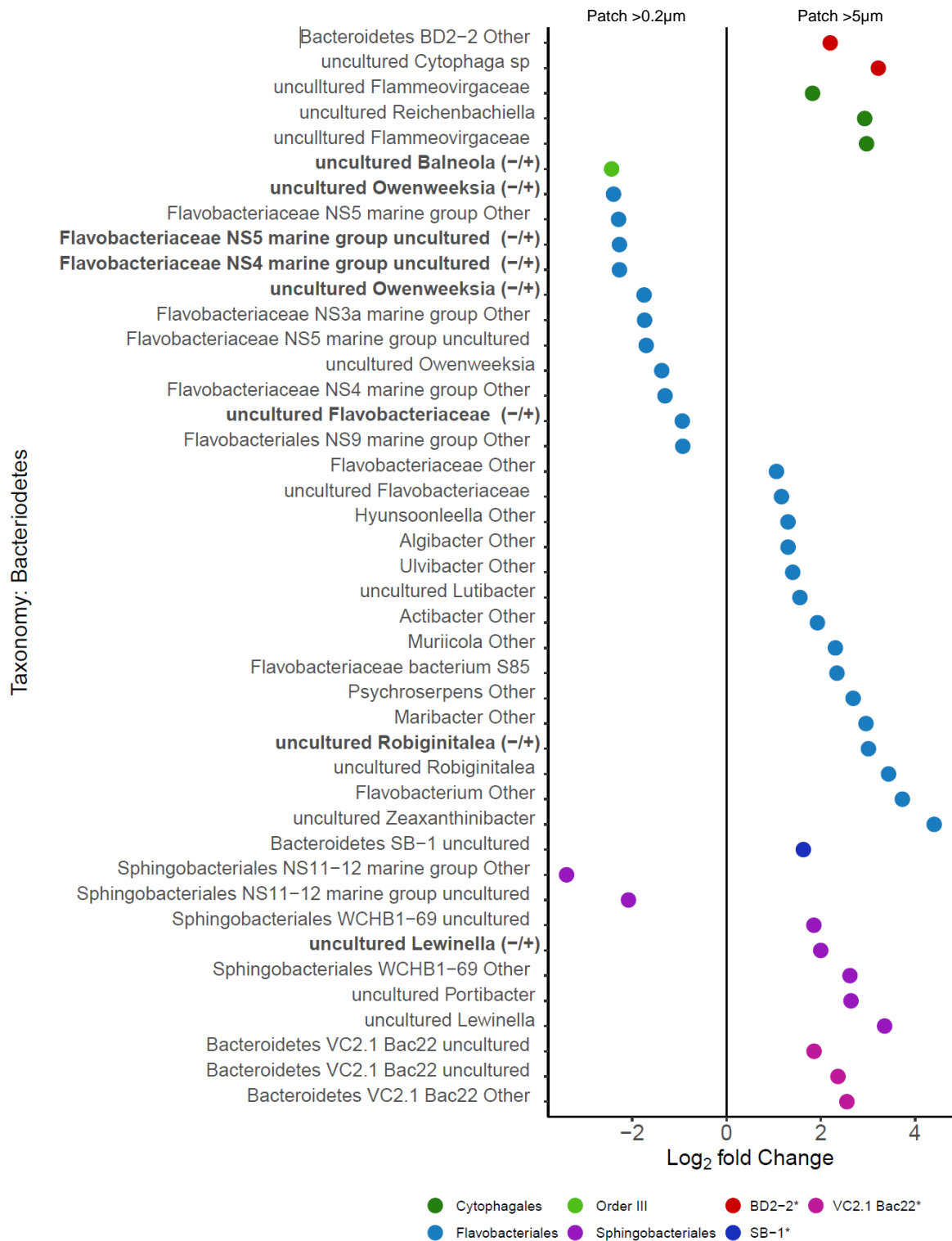

Supplement: S7 Fig — Negative log2 fold changes represent lineages enriched in >0.2μm samples, while positive log2 fold changes represent lineages enriched in >5μm samples. Lineages that are part of the >5μm fraction (+) and >0.2μm fraction (-) core microbiomes are in bold, and italicized if unique to a core microbiome. Only significant differential abundances (alpha <0.05) are shown. Data is grouped by phyla (taxonomy) and colored by order. Unknown orders are listed as next lowest known taxonomy and indicated with *. (PDF) [file pone.0223067.s011.pdf]

S8 Fig.

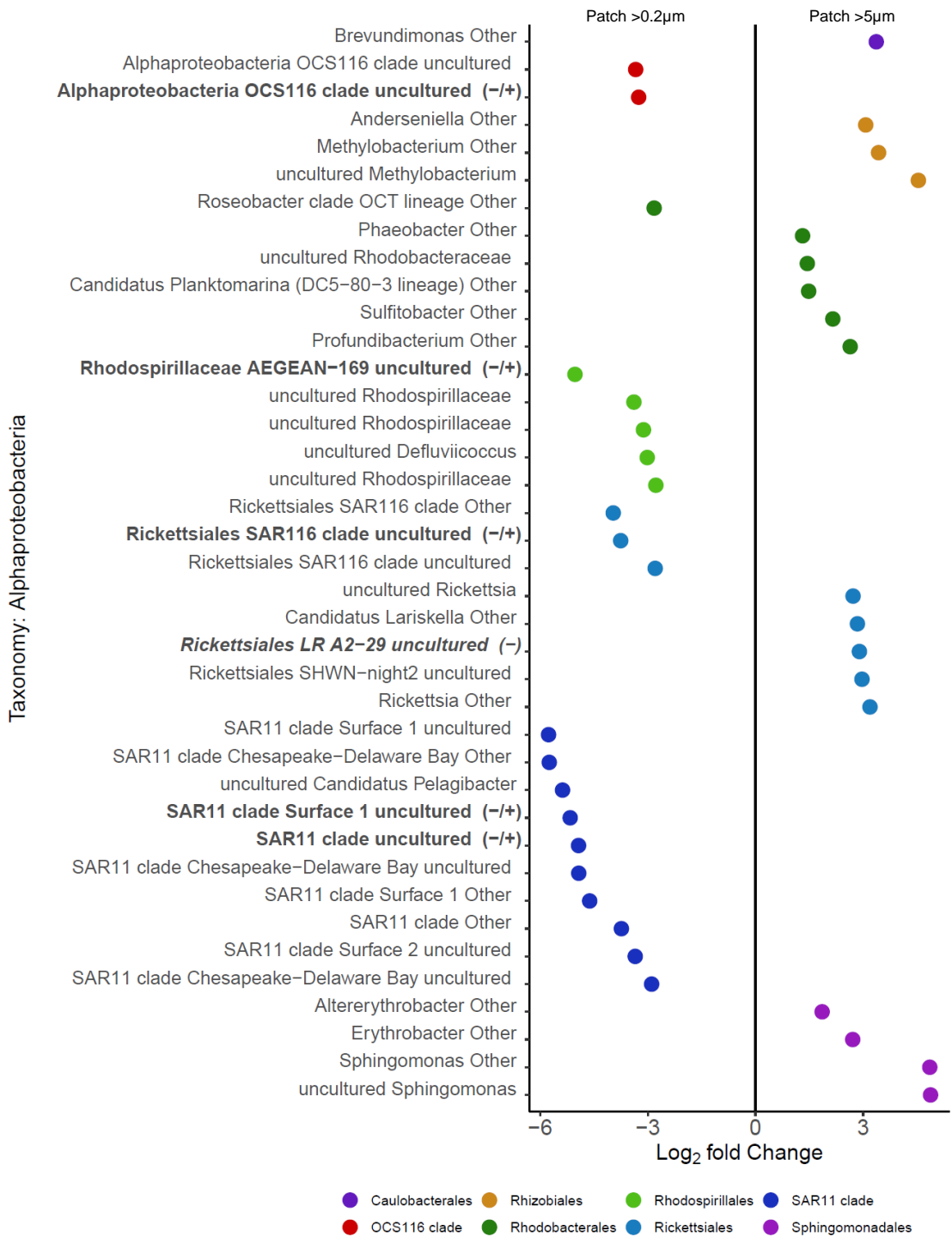

Supplement: S8 Fig — Negative log2 fold changes represent lineages enriched in >0.2μm samples, while positive log2 fold changes represent lineages enriched in >5μm samples. Lineages that are part of the >5μm fraction (+) and >0.2μm fraction (-) core microbiomes are in bold, and italicized if unique to a core microbiome. Only significant differential abundances (alpha <0.05) are shown. Data is grouped by phyla (taxonomy) and colored by order. Unknown orders are listed as next lowest known taxonomy and indicated with *. (PDF) [file pone.0223067.s012.pdf]

S9 Fig.

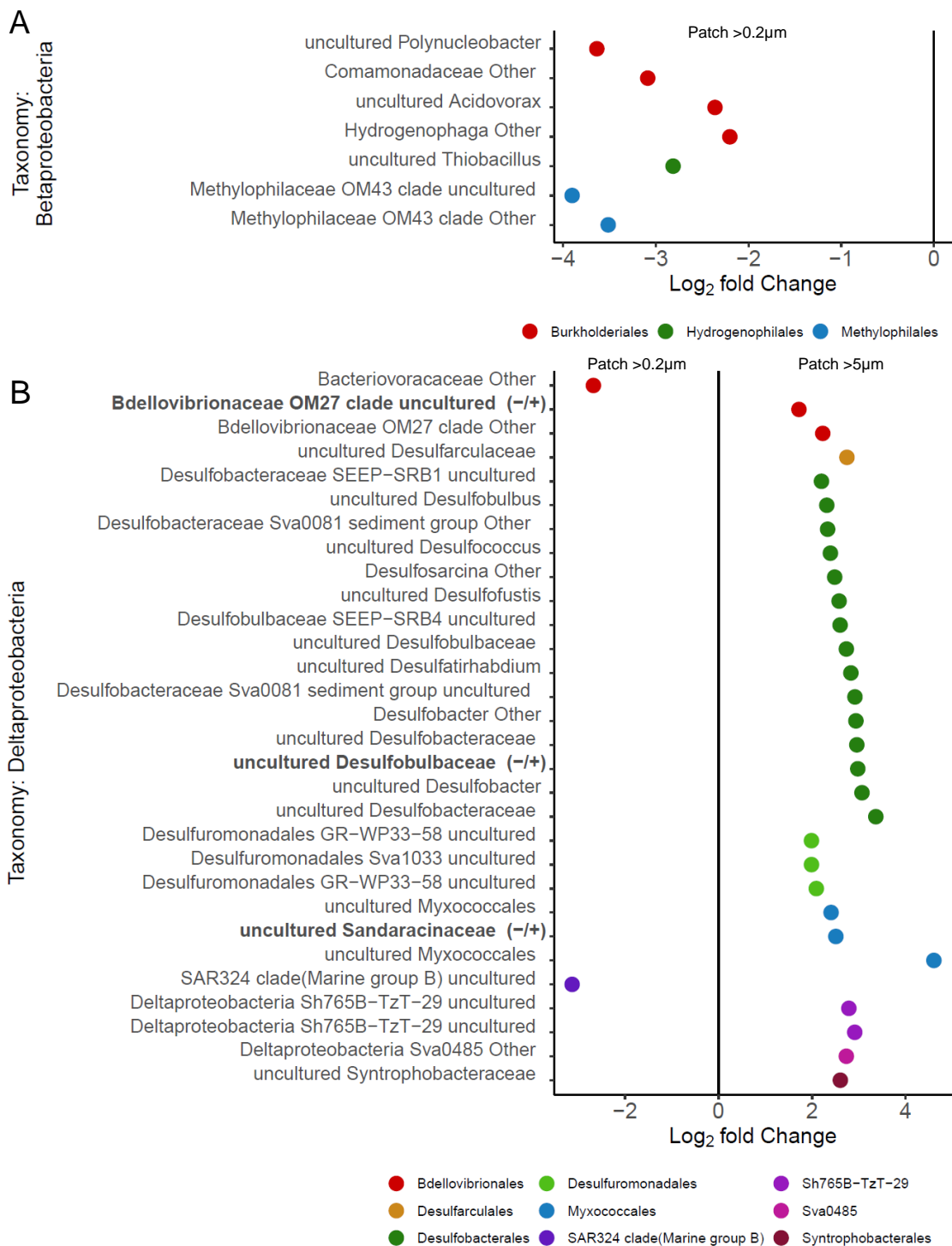

Supplement: S9 Fig — Differentially abundant 16S lineages among the Patch >0.2μm and Patch >5μm size fraction samples for A) Betaproteobacteria and B) Deltaproteobacteria. Negative log2 fold changes represent lineages enriched in >0.2μm samples, while positive log2 fold changes represent lineages enriched in >5μm samples. Lineages that are part of the >5μm fraction (+) and >0.2μm fraction (-) core microbiomes are in bold, and italicized if unique to a core microbiome. Only significant differential abundances (alpha <0.05) are shown. Data is grouped by phyla (taxonomy) and colored by order. Unknown orders are listed as next lowest known taxonomy and indicated with *. (PDF) [file pone.0223067.s013.pdf]

S10 Fig.

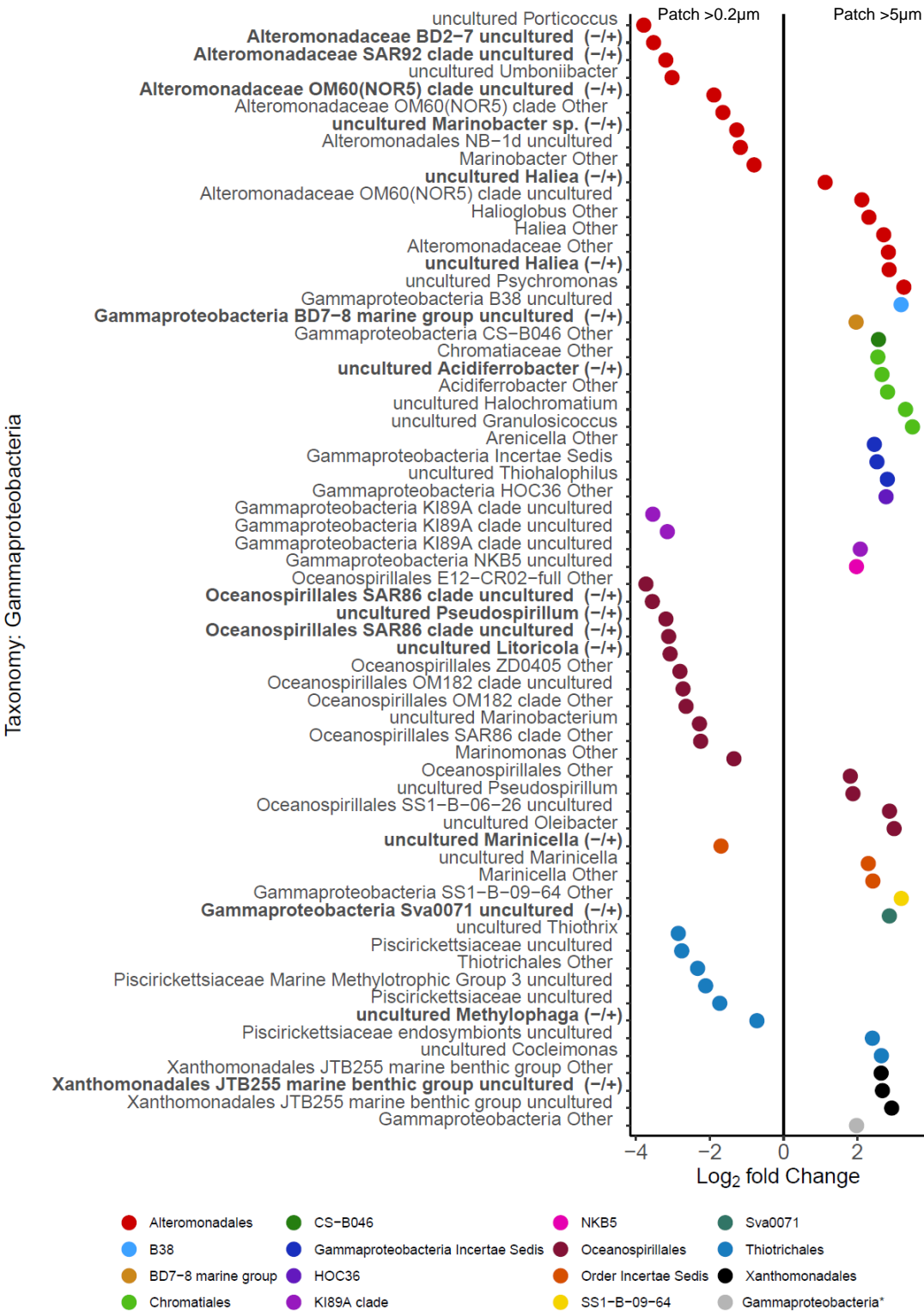

Supplement: S10 Fig — Negative log2 fold changes represent lineages enriched in >0.2μm samples, while positive log2 fold changes represent lineages enriched in >5μm samples. Lineages that are part of the >5μm fraction (+) and >0.2μm fraction (-) core microbiomes are in bold, and italicized if unique to a core microbiome. Only significant differential abundances (alpha <0.05) are shown. Data is grouped by phyla (taxonomy) and colored by order. Unknown orders are listed as next lowest known taxonomy and indicated with *. (PDF) [file pone.0223067.s014.pdf]

S11 Fig.

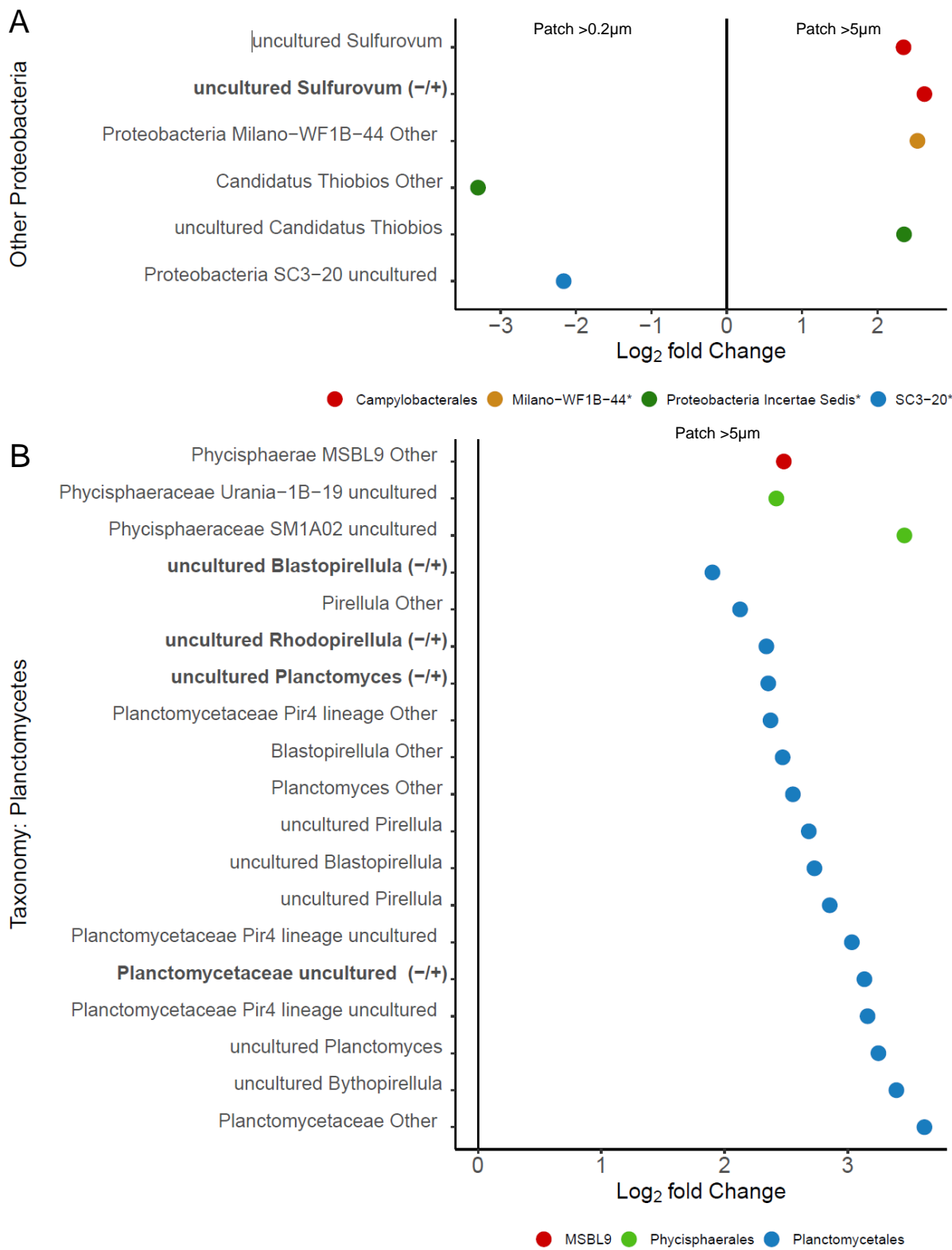

Supplement: S11 Fig — Differentially abundant 16S lineages among the Patch >0.2μm and Patch >5μm size fraction samples for A) other Proteobacteria and B) Planctomycetes. Negative log2 fold changes represent lineages enriched in >0.2μm samples, while positive log2 fold changes represent lineages enriched in >5μm samples. Lineages that are part of the >5μm fraction (+) and >0.2μm fraction (-) core microbiomes are in bold, and italicized if unique to a core microbiome. Only significant differential abundances (alpha <0.05) are shown. Data is grouped by phyla (taxonomy) and colored by order. Unknown orders are listed as next lowest known taxonomy and indicated with *. (PDF) [file pone.0223067.s015.pdf]

S12 Fig.

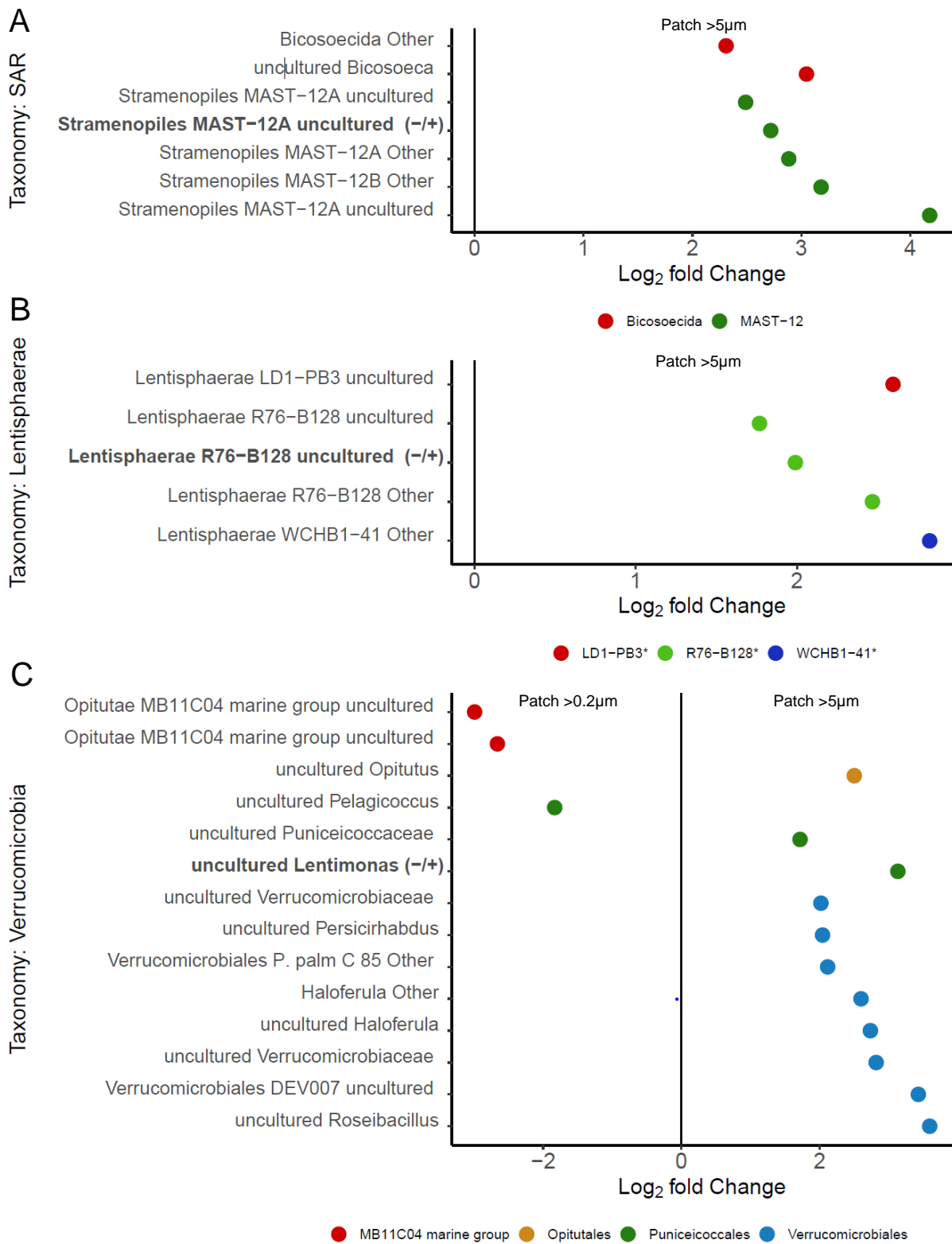

Supplement: S12 Fig — Differentially abundant 16S lineages among the Patch >0.2μm and Patch >5μm size fraction samples for A) SAR, B) Lentisphaerae and C) Verrucomicrobia. Negative log2 fold changes represent lineages enriched in >0.2μm samples, while positive log2 fold changes represent lineages enriched in >5μm samples. Lineages that are part of the >5μm fraction (+) and >0.2μm fraction (-) core microbiomes are in bold, and italicized if unique to a core microbiome. Only significant differential abundances (alpha <0.05) are shown. Data is grouped by phyla (taxonomy) and colored by order. Unknown orders are listed as next lowest known taxonomy and indicated with *. (PDF) [file pone.0223067.s016.pdf]

S13 Fig.

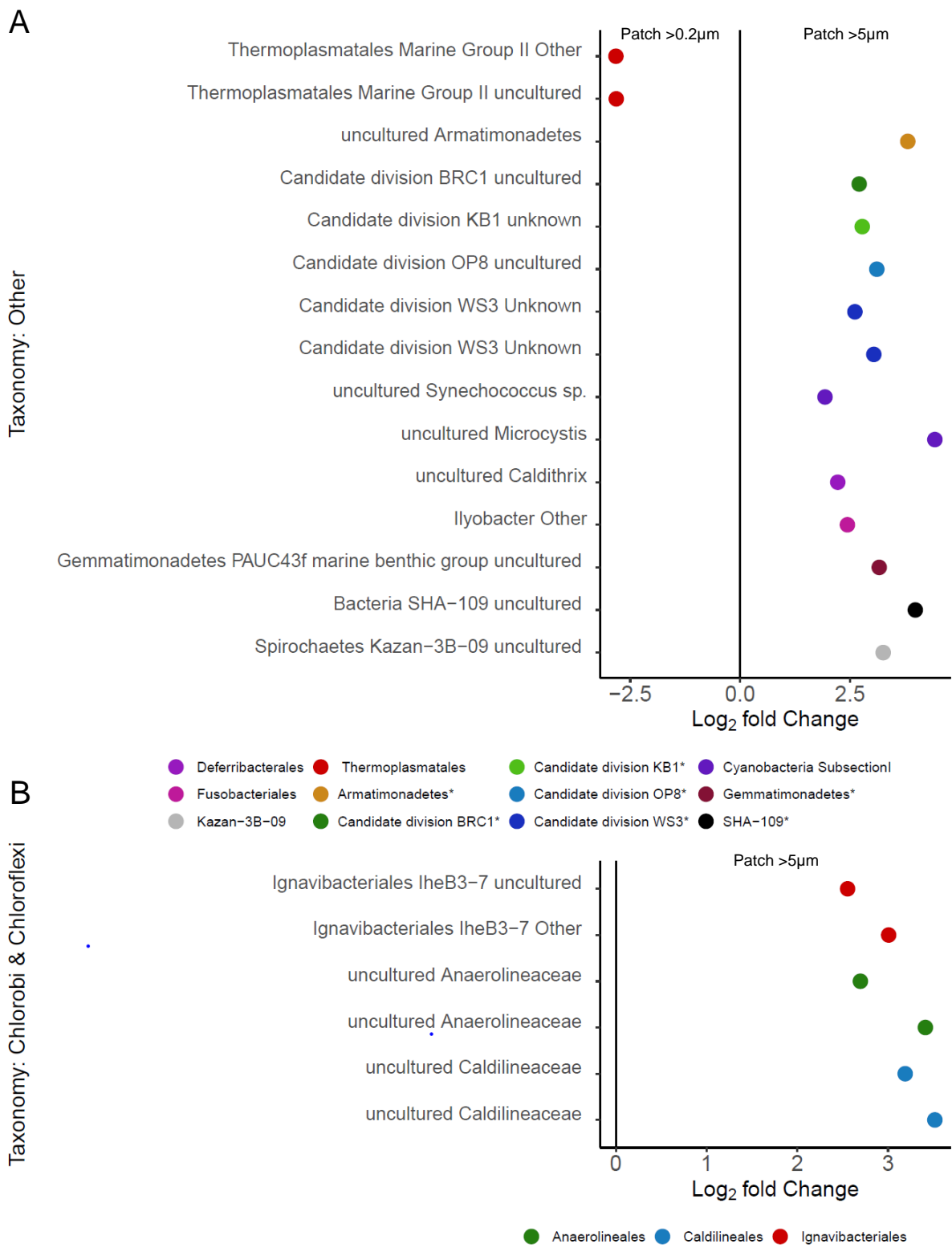

Supplement: S13 Fig — Differentially abundant 16S lineages among the Patch >0.2μm and Patch >5μm size fraction samples for A) Other and B) Chlorobi and Chloroflexi. Negative log2 fold changes represent lineages enriched in >0.2μm samples, while positive log2 fold changes represent lineages enriched in >5μm samples. Lineages that are part of the >5μm fraction (+) and >0.2μm fraction (-) core microbiomes are in bold, and italicized if unique to a core microbiome. Only significant differential abundances (alpha <0.05) are shown. Data is grouped by phyla (taxonomy) and colored by order. Unknown orders are listed as next lowest known taxonomy and indicated with *. (PDF) [file pone.0223067.s017.pdf]

S14 Fig.

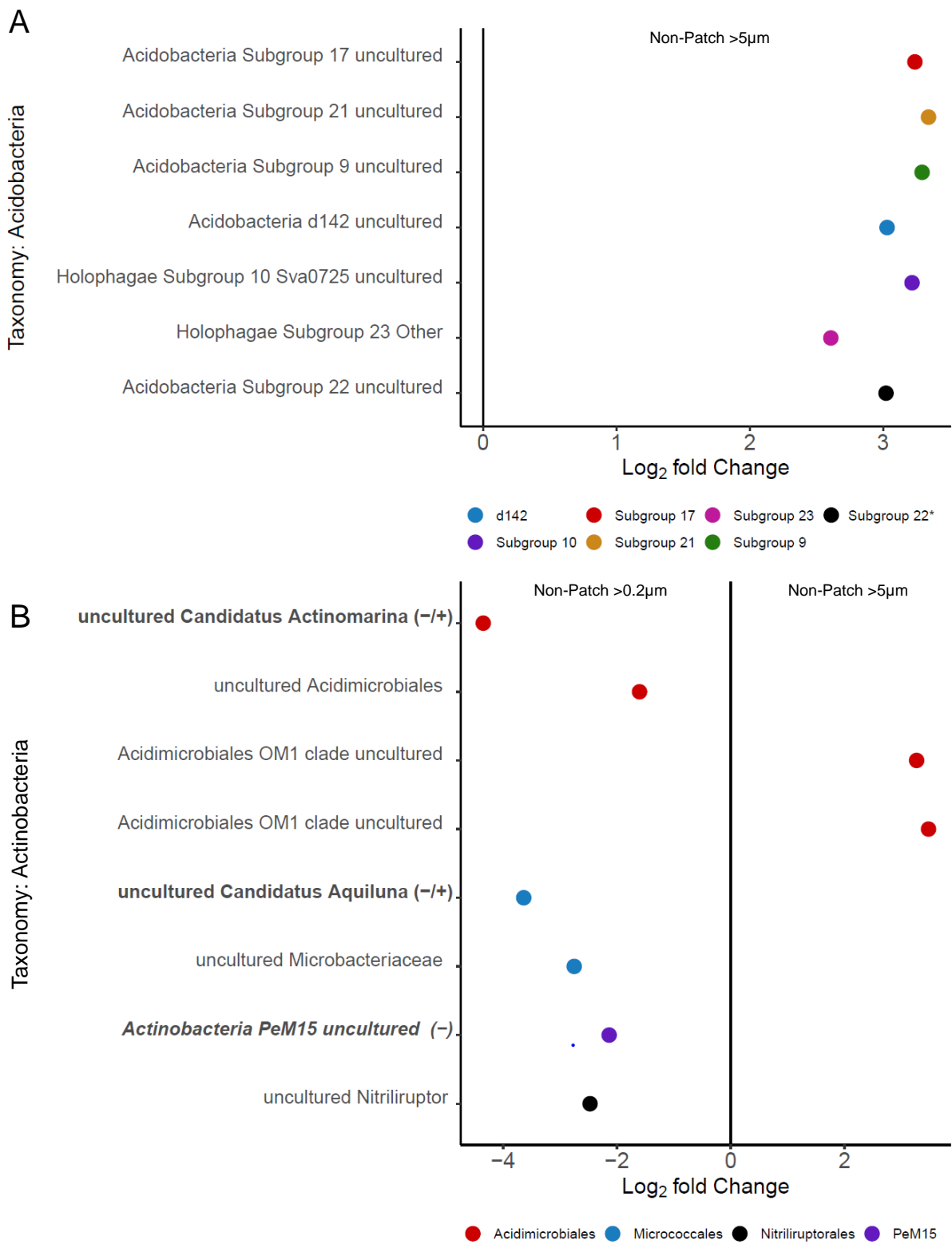

Supplement: S14 Fig — Differentially abundant 16S lineages among the Non-patch >0.2μm and Non-patch >5μm size fraction samples for A) Acidobacteria and B) Actinobacteria. Negative log2 fold changes represent lineages enriched in >0.2μm samples, while positive log2 fold changes represent lineages enriched in >5μm samples. Lineages that are part of the >5μm fraction (+) and >0.2μm fraction (-) core microbiomes are in bold, and italicized if unique to a core microbiome. Only significant differential abundances (alpha <0.05) are shown. Data is grouped by phyla (taxonomy) and colored by order. Unknown orders are listed as next lowest known taxonomy and indicated with *. (PDF) [file pone.0223067.s018.pdf]

S15 Fig.

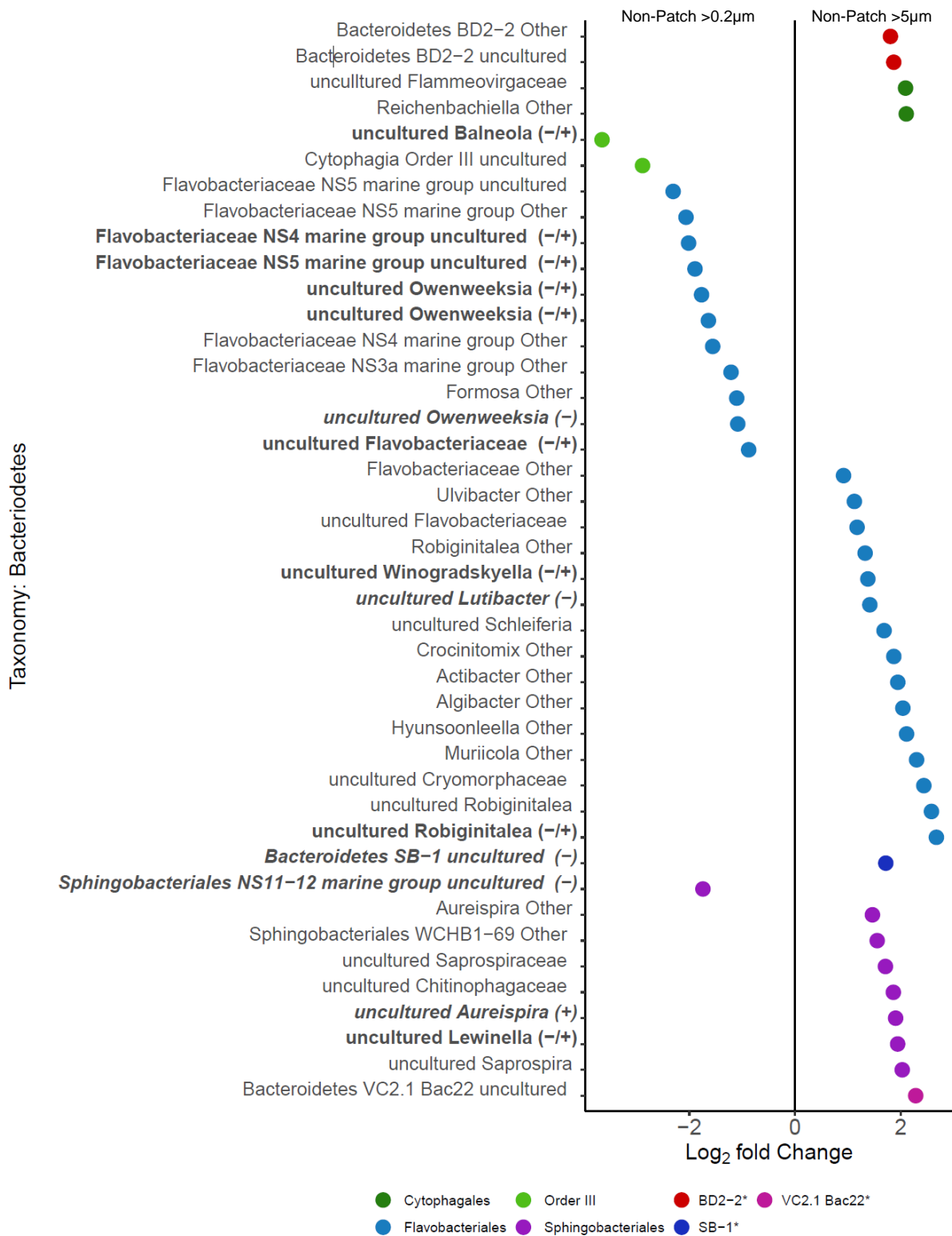

Supplement: S15 Fig — Negative log2 fold changes represent lineages enriched in >0.2μm samples, while positive log2 fold changes represent lineages enriched in >5μm samples. Lineages that are part of the >5μm fraction (+) and >0.2μm fraction (-) core microbiomes are in bold, and italicized if unique to a core microbiome. Only significant differential abundances (alpha <0.05) are shown. Data is grouped by phyla (taxonomy) and colored by order. Unknown orders are listed as next lowest known taxonomy and indicated with *. (PDF) [file pone.0223067.s019.pdf]

S16 Fig.

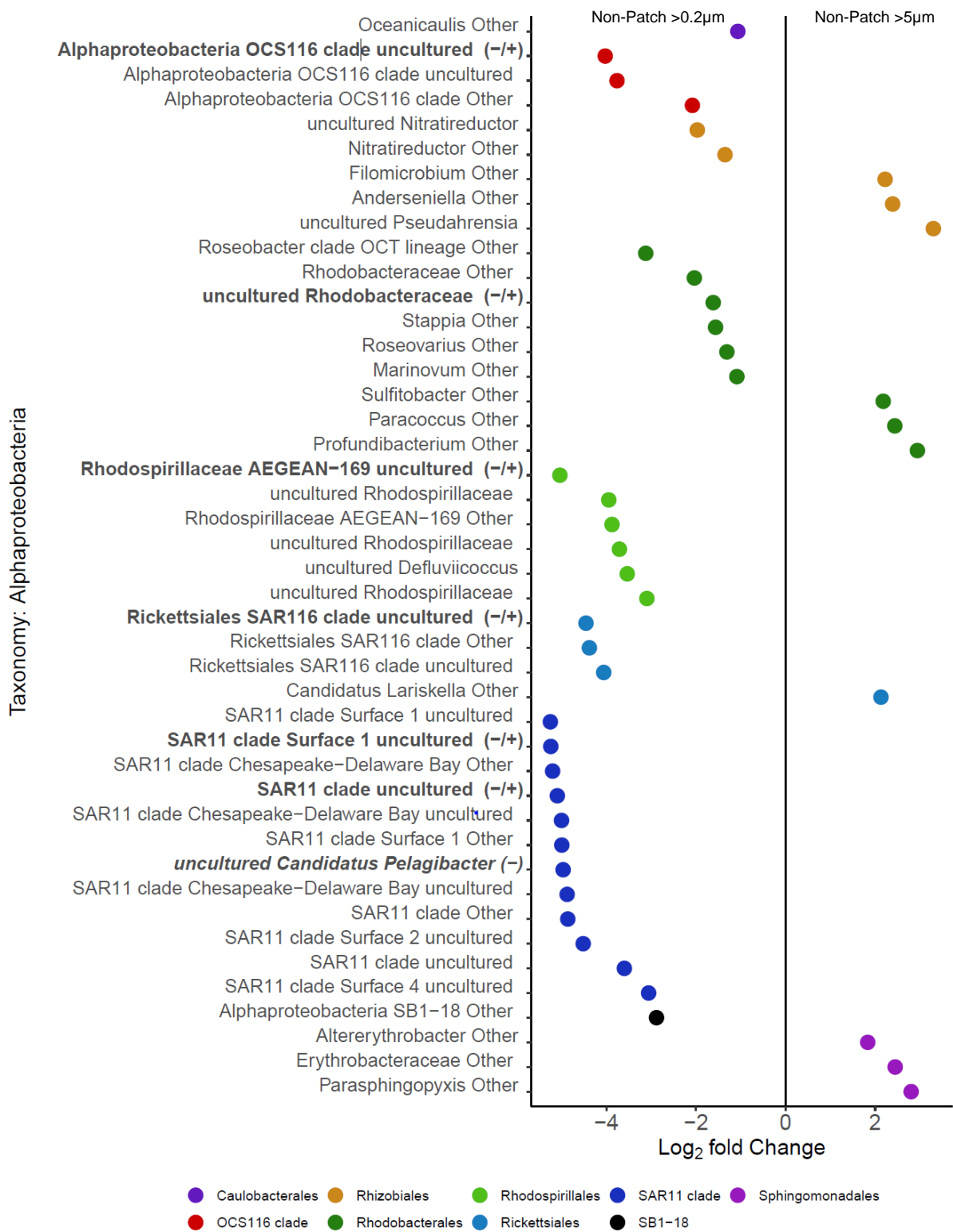

Supplement: S16 Fig — Negative log2 fold changes represent lineages enriched in >0.2μm samples, while positive log2 fold changes represent lineages enriched in >5μm samples. Lineages that are part of the >5μm fraction (+) and >0.2μm fraction (-) core microbiomes are in bold, and italicized if unique to a core microbiome. Only significant differential abundances (alpha <0.05) are shown. Data is grouped by phyla (taxonomy) and colored by order. Unknown orders are listed as next lowest known taxonomy and indicated with *. (PDF) [file pone.0223067.s020.pdf]

S17 Fig.

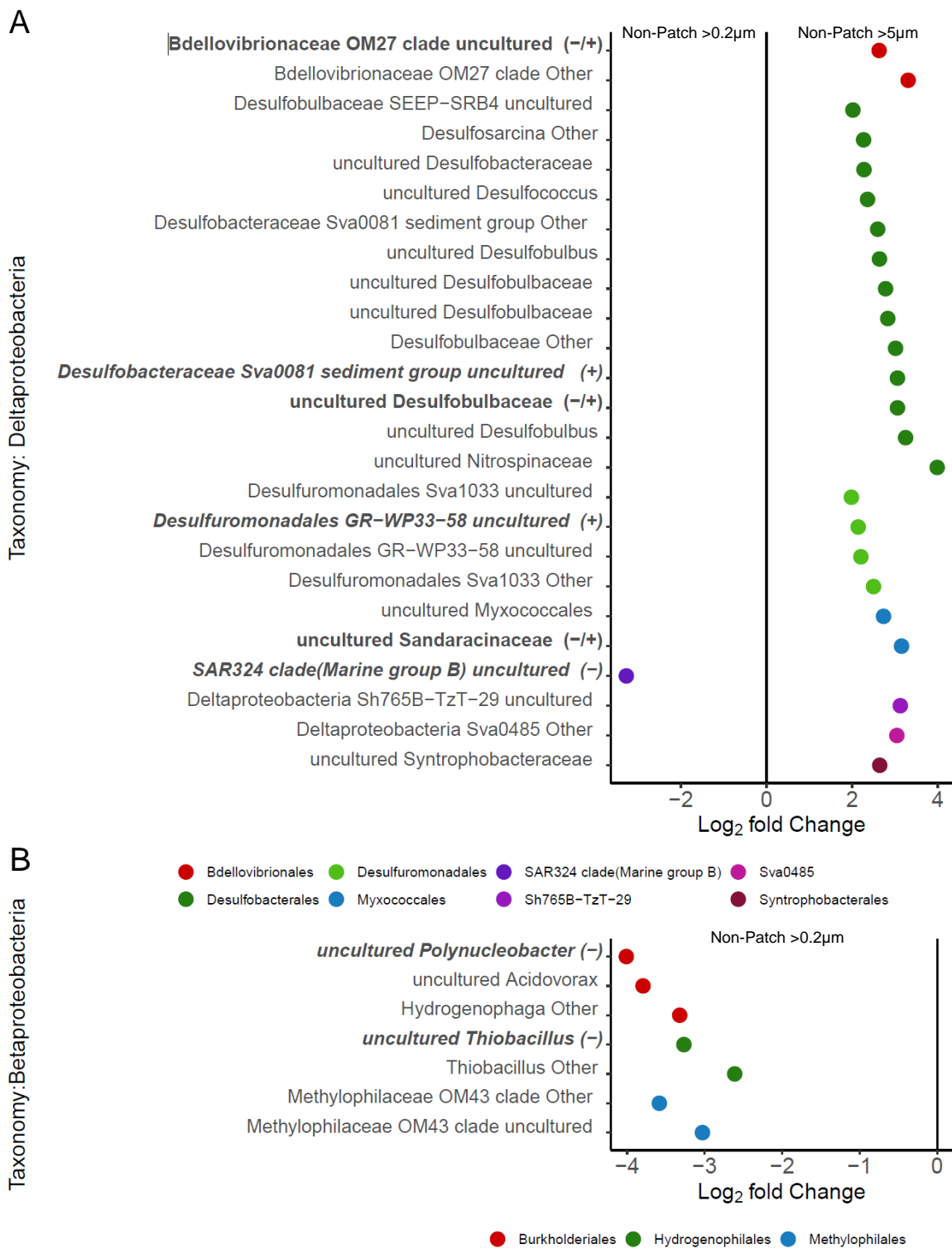

Supplement: S17 Fig — Differentially abundant 16S lineages among the Non-patch >0.2μm and Non-patch >5μm size fraction samples for A) Deltaproteobacteria and B) Betaproteobacteria. Negative log2 fold changes represent lineages enriched in >0.2μm samples, while positive log2 fold changes represent lineages enriched in >5μm samples. Lineages that are part of the >5μm fraction (+) and >0.2μm fraction (-) core microbiomes are in bold, and italicized if unique to a core microbiome. Only significant differential abundances (alpha <0.05) are shown. Data is grouped by phyla (taxonomy) and colored by order. Unknown orders are listed as next lowest known taxonomy and indicated with *. (PDF) [file pone.0223067.s021.pdf]

S18 Fig.

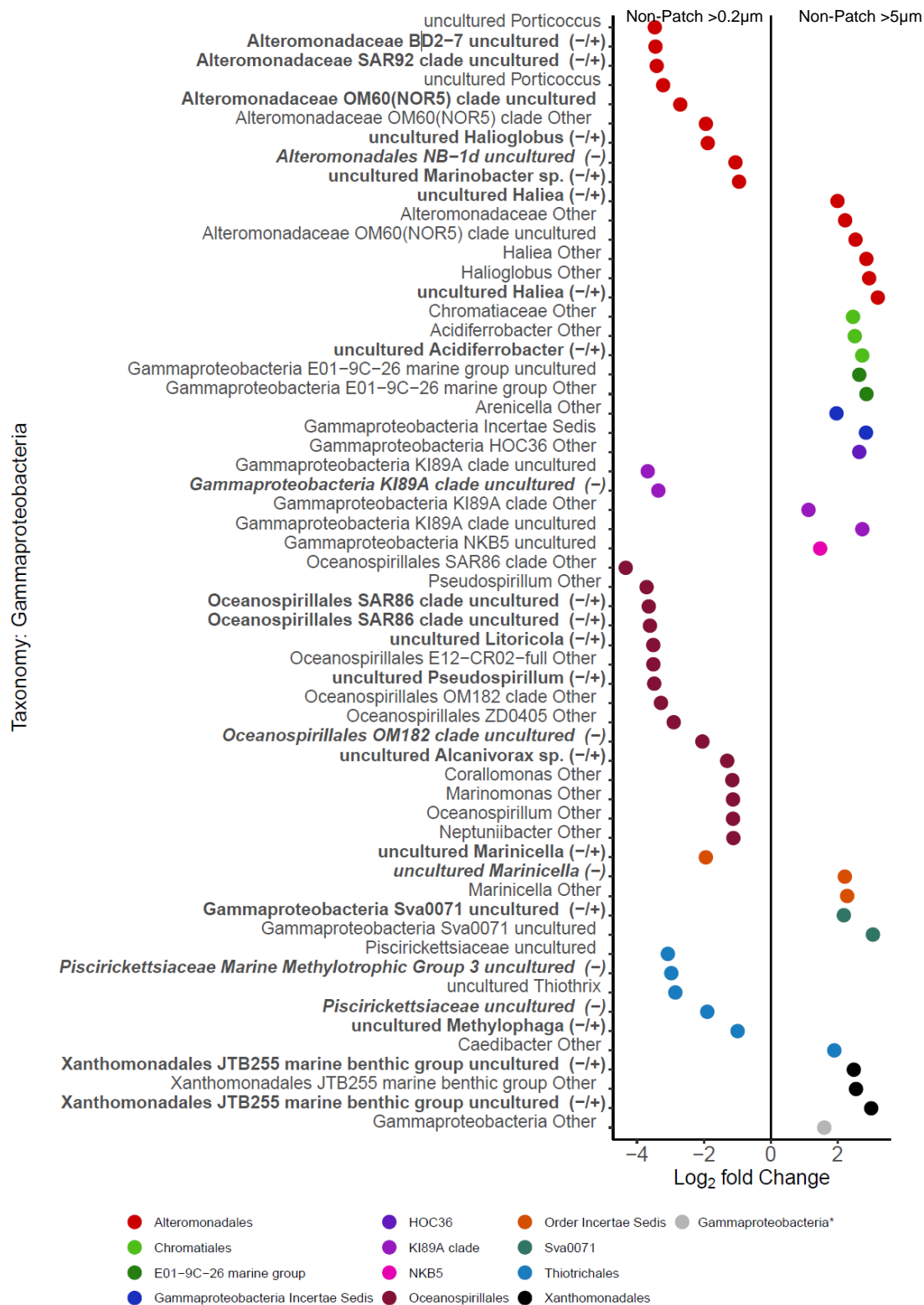

Supplement: S18 Fig — Negative log2 fold changes represent lineages enriched in >0.2μm samples, while positive log2 fold changes represent lineages enriched in >5μm samples. Lineages that are part of the >5μm fraction (+) and >0.2μm fraction (-) core microbiomes are in bold, and italicized if unique to a core microbiome. Only significant differential abundances (alpha <0.05) are shown. Data is grouped by phyla (taxonomy) and colored by order. Unknown orders are listed as next lowest known taxonomy and indicated with *. (PDF) [file pone.0223067.s022.pdf]

S19 Fig.

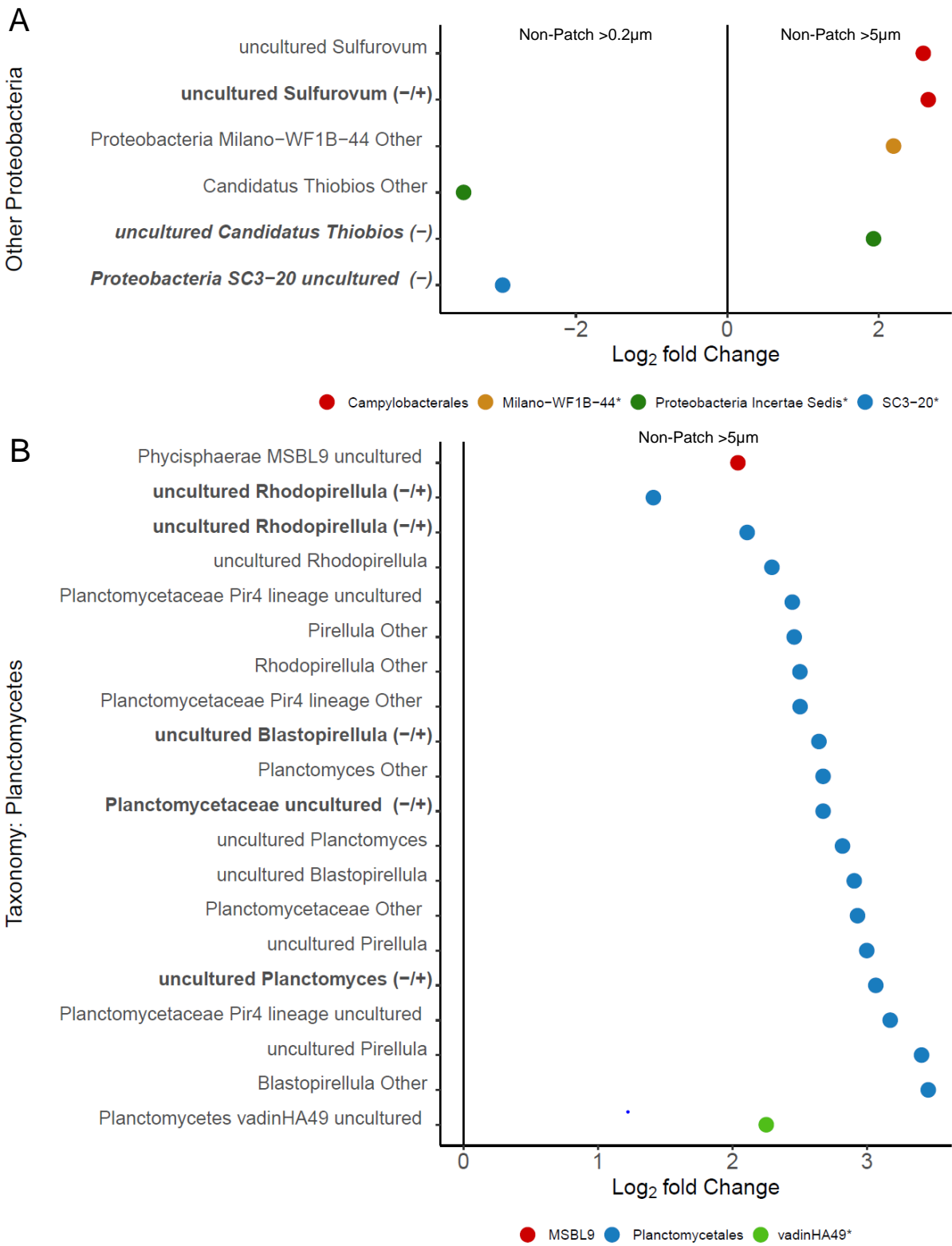

Supplement: S19 Fig — Differentially abundant 16S lineages among the Non-patch >0.2μm and Non-patch >5μm size fraction samples for A) other Proteobacteria and B) Planctomycetes. Negative log2 fold changes represent lineages enriched in >0.2μm samples, while positive log2 fold changes represent lineages enriched in >5μm samples. Lineages that are part of the >5μm fraction (+) and >0.2μm fraction (-) core microbiomes are in bold, and italicized if unique to a core microbiome. Only significant differential abundances (alpha <0.05) are shown. Data is grouped by phyla (taxonomy) and colored by order. Unknown orders are listed as next lowest known taxonomy and indicated with *. (PDF) [file pone.0223067.s023.pdf]

S20 Fig.

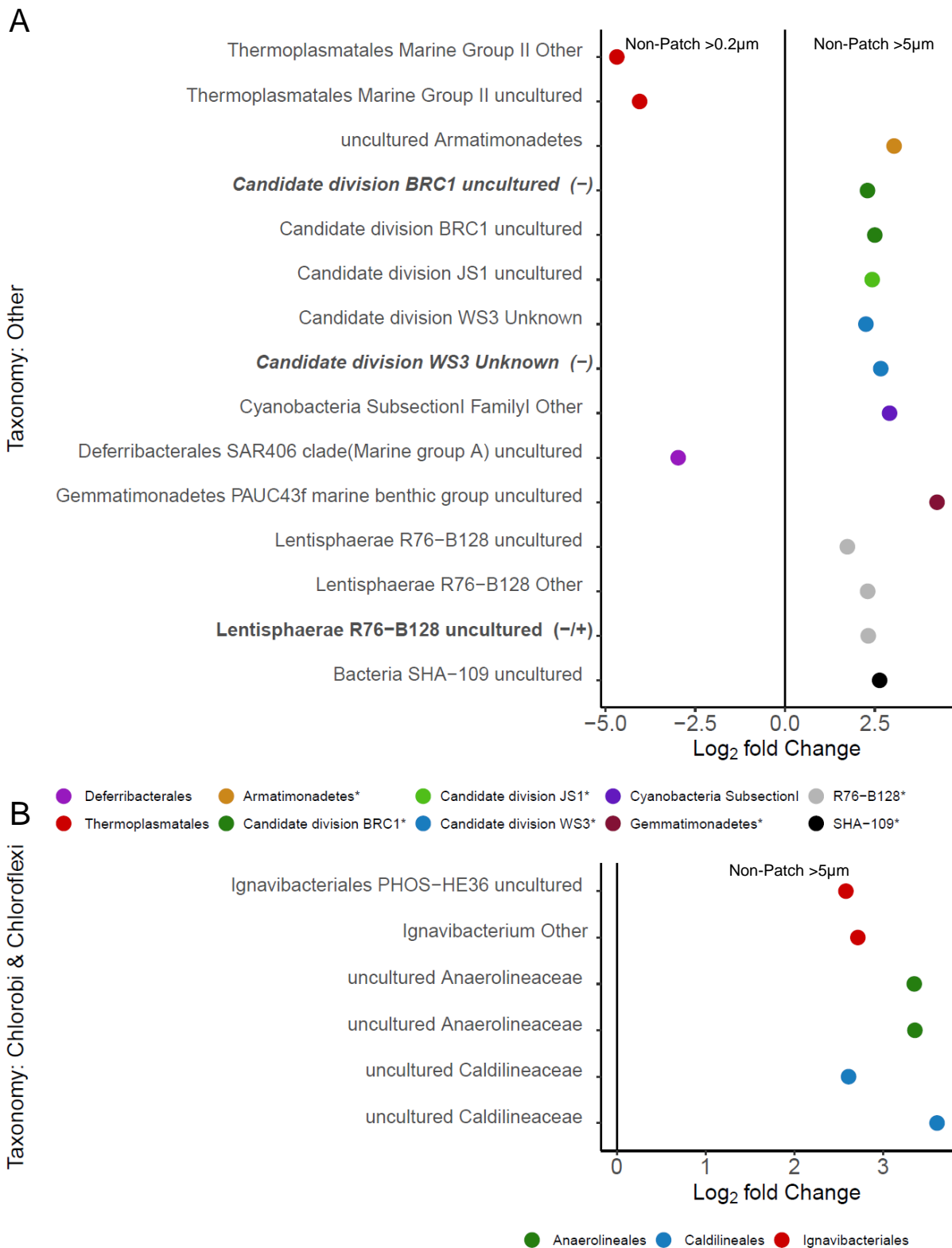

Supplement: S20 Fig — Differentially abundant 16S lineages among the Non-patch >0.2μm and Non-patch >5μm size fraction samples for A) Other and B) Chlorobi and Chloroflexi. Negative log2 fold changes represent lineages enriched in >0.2μm samples, while positive log2 fold changes represent lineages enriched in >5μm samples. Lineages that are part of the >5μm fraction (+) and >0.2μm fraction (-) core microbiomes are in bold, and italicized if unique to a core microbiome. Only significant differential abundances (alpha <0.05) are shown. Data is grouped by phyla (taxonomy) and colored by order. Unknown orders are listed as next lowest known taxonomy and indicated with *. (PDF) [file pone.0223067.s024.pdf]

S21 Fig.

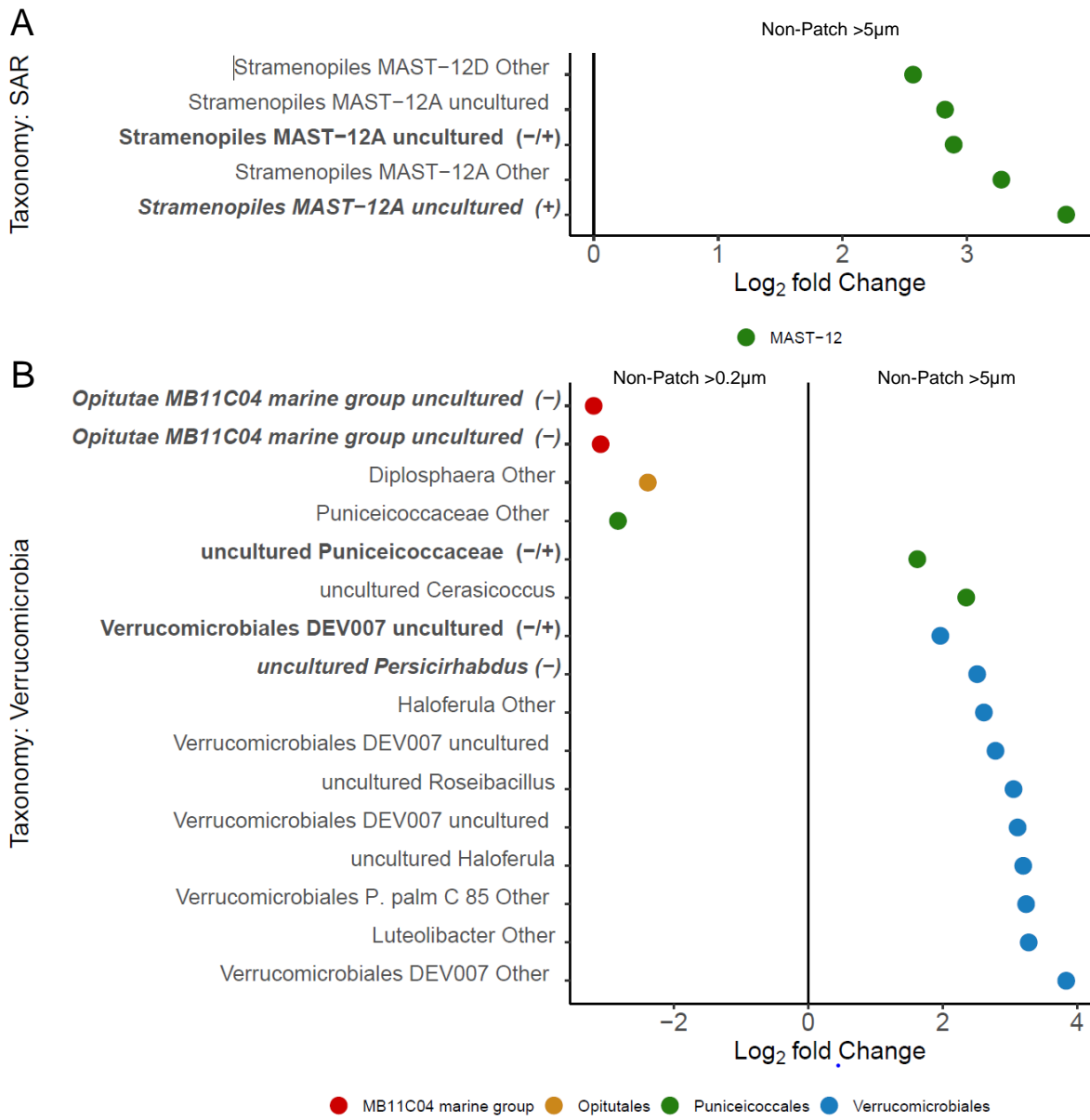

Supplement: S21 Fig — Differentially abundant 16S lineages among the Non-patch >0.2μm and Non-patch >5μm size fraction samples for A) SAR and B) Verrucomicrobia. Negative log2 fold changes represent lineages enriched in >0.2μm samples, while positive log2 fold changes represent lineages enriched in >5μm samples. Lineages that are part of the >5μm fraction (+) and >0.2μm fraction (-) core microbiomes are in bold, and italicized if unique to a core microbiome. Only significant differential abundances (alpha <0.05) are shown. Data is grouped by phyla (taxonomy) and colored by order. Unknown orders are listed as next lowest known taxonomy and indicated with *. (PDF) [file pone.0223067.s025.pdf]

S22 Fig.

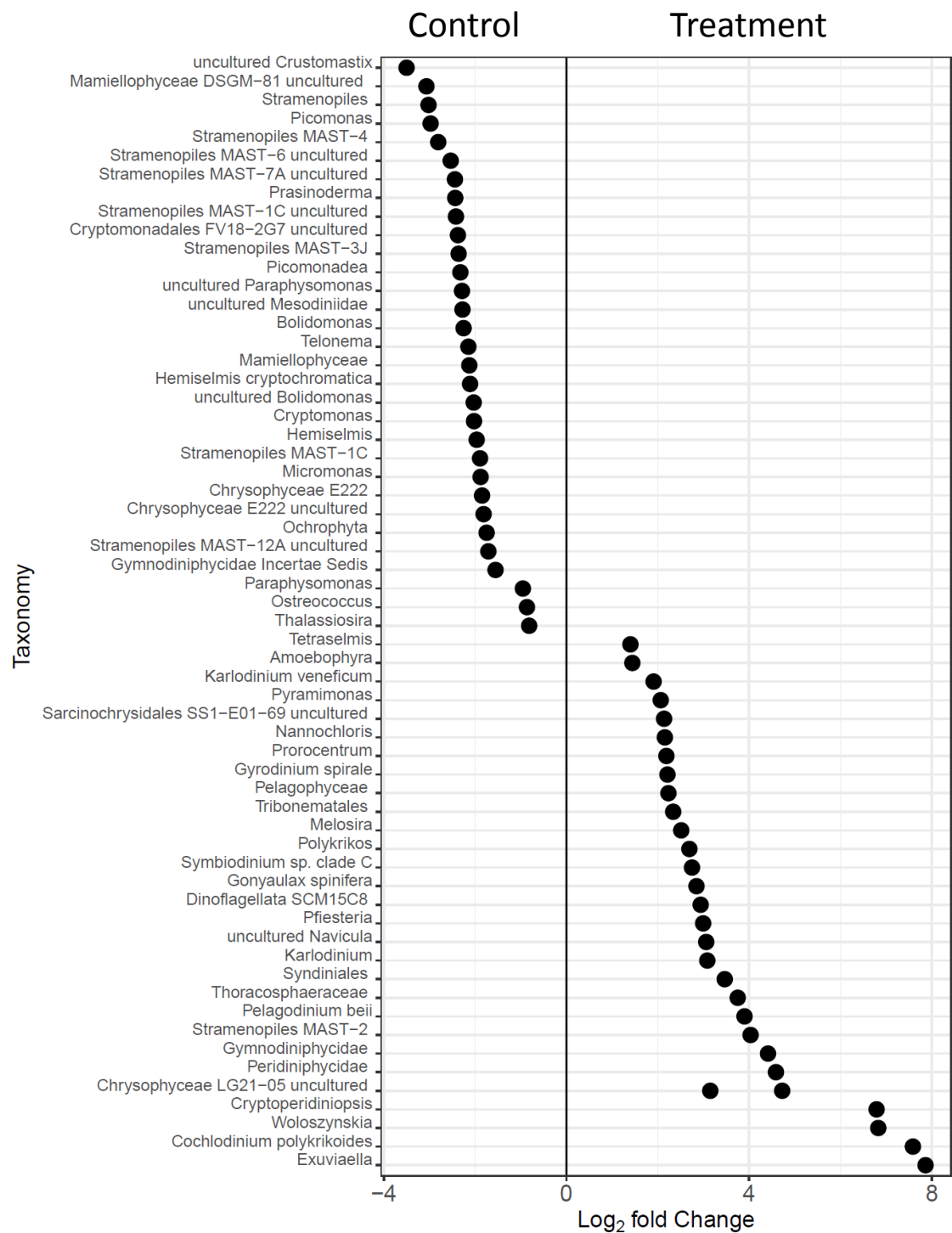

Supplement: S22 Fig — Negative log2 fold changes represent lineages enriched in control samples, while positive log2 fold changes represent lineages enriched in treatment samples. (PDF) [file pone.0223067.s026.pdf]
